# Role of Sulfate Transporters in Chromium Tolerance in *Scenedesmus acutus* M. (Sphaeropleales)

**DOI:** 10.3390/plants11020223

**Published:** 2022-01-15

**Authors:** Michele Ferrari, Radiana Cozza, Matteo Marieschi, Anna Torelli

**Affiliations:** 1 Department of Biology, Ecology and Earth Science, University of Calabria, Ponte P. Bucci, Arcavacata di Rende, 87036 Cosenza, Italy; michele.ferrari@unical.it; 2 Department of Chemistry, Life Sciences and Environmental Sustainability, University of Parma, Viale delle Scienze 11/A, 43124 Parma, Italy; matteo.marieschi@unipr.it

**Keywords:** abiotic stress, chromium tolerance, heavy metals, microalgae sulfur metabolism, *Scenedesmus acutus*, sulfate-starvation, sulfate transporters, sulfur uptake

## Abstract

Sulfur (S) is essential for the synthesis of important defense compounds and in the scavenging potential of oxidative stress, conferring increased capacity to cope with biotic and abiotic stresses. Chromate can induce a sort of S-starvation by competing for uptake with SO_4_^2−^ and causing a depletion of cellular reduced compounds, thus emphasizing the role of S-transporters in heavy-metal tolerance. In this work we analyzed the sulfate transporter system in the freshwater green algae *Scenedesmus acutus*, that proved to possess both H^+^/SO_4_^2−^ (SULTRs) and Na^+^/SO_4_^2−^ (SLTs) plasma membrane sulfate transporters and a chloroplast-envelope localized ABC-type holocomplex. We discuss the sulfate uptake system of *S. acutus* in comparison with other taxa, enlightening differences among the clade Sphaeropleales and Volvocales/Chlamydomonadales. To define the role of S transporters in chromium tolerance, we analyzed the expression of *SULTRs* and *SULPs* components of the chloroplast ABC transporter in two strains of *S. acutus* with different Cr(VI) sensitivity. Their differential expression in response to Cr(VI) exposure and S availability seems directly linked to Cr(VI) tolerance, confirming the role of sulfate uptake/assimilation pathways in the metal stress response. The *SULTRs* up-regulation, observed in both strains after S-starvation, may directly contribute to enhancing Cr-tolerance by limiting Cr(VI) uptake and increasing sulfur availability for the synthesis of sulfur-containing defense molecules.

## 1. Introduction

In photosynthetic organisms, the capacity to cope with biotic and abiotic stresses is supported by a process known as Sulfur Enhanced Defense (SED) [1], that relies on sulfur availability. End-products of the sulfur assimilation pathway, cysteine (Cys) and reduced glutathione (GSH), are involved in counteracting the negative impacts of different stressors. Among nonessential toxic agents for plant development, Chromium (Cr) is a common heavy metal, and a cause of environmental pollution, that has gained substantial consideration worldwide for its high levels in water and soil due to both natural and anthropogenic activities [2]. Cr(VI) usually occurs as chromate (CrO^4−^) and dichromate (Cr_2_O_4_^2−^) anionic forms and can easily cross cell membranes with active transport mechanisms [3,4] through nonspecific anion channels [5]. Interactions between chromium and sulfur in biological systems are numerous and somewhat contrasting; while S enhances cellular response to Cr, this can induce a sort of S-starvation, both through a competition for sulfate transporters and through a depletion of sulfur reduced compounds like Cys and GSH [6,7,8,9]. These thiol containing molecules are partly involved in metal chelation and partly oxidized to reduce ROS damages. On the other hand, Cys and GSH can also contribute to increasing chromium toxicity through the reduction of Cr(VI) to Cr(V)-Cr(III), incrementing ROS production and contributing to generation of the more genotoxic chromium form [8]. Cr(VI), besides competing for sulfate transporters, can also compete for the enzymes of the sulfate assimilation pathway [6,10,11,12,13,14,15], causing a decrease in Cys and methionine production and leading to a mistranslation of important proteins, once again mimicking an effect of S-starvation.

Sulfate transporters assume crucial importance in Cr(VI) tolerance, not only in regulating sulfur availability, but also in regulating chromium uptake, since chromate (CrO_4_^2−^) and sulfate (SO_4_^2−^) can compete for the same transporters due to the chemical similarity of the two anions. Algae plasma membrane transporters can couple the influx of sulfate with the cotransport of positively charged counterions such as protons (H^+^) and sodium ions (Na^+^). For these mechanisms, the counterion concentration gradients serve as the driving force for the influx of sulfate across the membranes [16]. The H^+^/SO_4_^2−^ transporters, namely the SULTR gene family (SULfate TRansporter), have been identified in all photosynthetic organisms studied so far [16,17,18]. SLT transporters (Sac1 Like Transporters) catalyze Na^+^/SO_4_^2−^ cotransport and share high similarity with bacteria, nonvascular plants and mammals’ sulfate transporters [17].

The SULTR and SLT transporters have different characteristics. The SULTR transporters belong to the SLC26 transporter class and possess 10–14 predicted transmembrane domains and, at the cytosolic carboxy-terminal end, a STAS (Sulfate Transporter and Anti-Sigma factor antagonist) domain, which is common in anion transporters and is believed to have regulatory functions [17]. The STAS domain is connected to the transmembrane portion through a poorly conserved region of variable length, known as the linker domain [17]. No crystal structure or electron diffraction structure for the SLC26 transmembrane domain has been reported, but several studies in different organisms support a homo-oligomeric or, more specifically, dimeric or tetrameric structures for these transporters [19]. Studies on *Arabidopsis thaliana* indicate that the STAS domain is essential for targeting the protein to plasmalemma, and affects both transport kinetics and protein stability [20]. Moreover, the STAS domain binds and activates the enzyme O-acetylserinelyase/Cysteine synthase (OASTL), whose coexpression downregulates SULTR1;2-mediated SO_4_^2−^ uptake [20].

SULTR-like sequences have been found in the diatoms *Thalassiosira pseudonana* and *Phaeodactylum tricornutum*, in the Pelagophyte *Aureococcus anophagefferens*, in the Rhodophyte *Cyanidioschizon merolae*, in the Prasinophycean *Ostreococcus* sp., as well as in the Chlorophycean green alga *Chlamydomonas reinhardtii*. Some of them contain STAS domains; however, the exact function of the products of these genes needs to be confirmed [21].

Na^+^/SO_4_^2−^ symporters, namely SLTs (Sac1 Like Transporters), were identified in *C. reinhardtii* mining the alga genome [22] searching for homologies with sequences of plant, animal and bacterial sulfate transporters [23]. These Na^+^/SO_4_^2−^ transporters are very similar to members of the SLC13 transporter family. *Chlamydomonas* members of the SLT family (SLT1, SLT2 and SLT3) contain 10–12 predicted transmembrane domains and an intracellular loop containing a TrkA-C domain. The TrkA-C domain is supposed to be involved in the regulation of the sulfate transporter activity but its function is not completely understood [23].

Green algae possess a variable number of SLT and SULTR transporters. In *C. reinhardtii* six putative plasma membrane sulfate transporters have been identified: three plant type H^+^/SO_4_^2−^ co-transporters, (SULTR1, SULTR2, SULTR3) and three animal-type Na^+^/SO_4_^2−^ co-transporters (SLT1 to SLT3) [23]. *Volvox carteri*, apparently, has only one H^+^/SO_4_^2−^ and two Na^+^/SO_4_^2−^ exchangers, while *Ostreococcus tauri* possesses two Na^+^/SO_4_^2−^ and no H^+^/SO_4_^2−^ transporters. Interestingly, no Cl^−^, HCO_3_^−^ or I^−^ cotransporters have been found in algae, although they exist in yeasts, fungi and animals [16]. Among the transporters belonging to both groups there are members with different affinity for the substrate and different inducibility. In *C. reinhardtii*, the transcripts of SULTR2, SLT1 and SLT2 increased upon S deprivation, while the transcript of SULTR1 suffered a drastic decrease, while SULTR3 and SLT3 remained unchanged [23]. Unfortunately, information on marine algal sulfate transporters is very poor, however, both types of transporters were found in *Emiliana huxleyi* genome [21].

In the inner chloroplast envelopment of green algae, sulfate transport is catalyzed by an ATP-binding cassette (ABC) transporter similar to that found in cyanobacteria [16,24,25]. This ABC transporter is a permease holocomplex formed by two heterodimers in which the proteins SulP and SulP2 (sulfate permease) form the transmembrane channel; each of them is bound to a sulfate-binding protein (Sbp) on the cytosolic side and to an ATP-binding protein (Sabc) (energizing the transport) on the stromal side [24]. Genes encoding for these proteins are similar to the CysT, CysW, SBPA and CysA genes of prokaryotes and have different distributions between nuclear and chloroplast genomes of different organisms [24,25]. In the red alga *C. merolae*, SulP and SulP2 are encoded by chloroplast genes, and Sabc and SBP by nuclear genes. Among green algae, a nonuniform distribution was also reported [24]. In the Chlorophyceae *C. reinhardtii*, all the four genes have indeed nuclear localization, while in the Trebouxiophyceae *Chlorella vulgaris* and in the Prasinophyceae *Nephroselmis olivacea*, SulP and Sabc are encoded by chloroplast genome, whereas *SulP1* and *SBP* are presumably nuclear genes because of their absence in chloroplast genomes [25]. Similar distributions were observed in the Charophyceae *Chlorokybus atmophyticus*, *Mesostigma viride*, *Zygnema circumcarinatum* as well as in the liverworts *Marchantia polymorpha* and in the Anthocerota *Anthoceros formosa* [25]. No such transporters have been identified in other organisms [16], and the mechanism of plastidial sulfate transport in vascular plants is still not known [25].

Unfortunately, the relatively detailed understanding of sulfate uptake in *Chlamydomonas* cannot be simply transferred to other microalgae. Different behaviors may be expected between freshwater and marine algae since sulfate concentration in the oceans is quite high and constant, and the presence of a finely regulated system may be unnecessary [26].

Many studies have shown that a difference in sulfur metabolism can be the basis of the different Cr(VI) sensitivity in two strains of the freshwater green alga *Scenedesmus acutus* (Chlorophyceae) [27,28,29,30]. Moreover, these studies further indicated that after a period of S-deprivation, a transient increase in chromium tolerance occurred both in the wild-type (wt) and in the Cr-tolerant strain (Cr-t), probably as a consequence of the enhancement of the entire sulfur assimilation pathway. After medium renewal following S-starvation, S-replete cells of both strains showed an S content significantly higher than in S-sufficient cells. Moreover, the observed increase was significantly higher in the Cr-t with resulting in greater Cys and GSH production in this strain [27,28,29,30]. Evidence of a change in the sulfate uptake system was further observed in this freshwater alga upon S-starvation [30].

Taking advantage of the availability of these two strains with different sensitivities to Cr(VI) and sulfur deprivation, the aim of the present study was to investigate the role of sulfate transporters in Cr(VI) tolerance. To this end, the expression of SULTR plasma membrane and the chloroplast ABC transporters was analyzed in the two strains, just after 3-day preculture in standard and S deprived medium and during recovery in sulfate-supplemented medium, in the presence of Cr(VI).

The two strains and their different response to Cr(VI) toxicity and S-starvation may constitute a good model for elucidating the mechanisms that link chromium tolerance and sulfur availability.

## 2. Results

In our previous studies we identified the sequences of several genes related to sulfur uptake/assimilation [31] and demonstrated that the Cr-t strain is characterized by a different uptake of both S and Cr(VI) [30]. This prompted us to analyze the genes with a role in the transport of sulfur across the plasma-membrane as well as in the uptake in the chloroplast.

### 2.1. Analysis of Plasma-Membrane Sulfate Transporter Genes

#### 2.1.1. SaSULTRs-Type Transporters

Two genes encoding putative SULTR-type transporters (H^+^/SO_4_^2−^), previously identified [31] were analyzed in this work, namely *SaSULTR1* and *SaSULTR2*.

**Figure 1:**
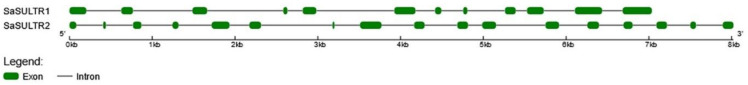
Structures of two *SaSULTRs* genes.

Blasting these sequences on the *Tetradesmus obliquus* UTEX393 genome (available in NCBI database), we found the presence of two distinct loci, one on scaffold 1191 corresponding to the sequence designated as *SaSULTR1*, and the other corresponding to *SaSULTR2*, located on scaffold 86. Their deduced amino acid sequences are very similar (48% identity and 63% similarity sequence), but the gene structure of *SaSULTR1* and *SaSULTR2* is different (Figure 1).

The *SaSULTR1* gene is organized in 12 exons separated by 11 introns, whereas *SaSULTR2* gene structure is characterized by 15 exons with 14 introns interposed (Figure 1). Both intron sizes and positions are different between the two genes as are the derived coding sequences (2133 and 1903 bp for *SaSULTR1* and *SaSULTR2*) for predicted polypeptides of 711 and 641 amino acids.

#### 2.1.2. SLTs-Type Transporters

Two genes, named Sa*SLTa* and *SaSLTb*, homologous to SLT-type transporters (Na^+^)/SO_4_^2−^), have been previously identified [31]. The mapping of these sequences on the *T. obliquus* UTEX393 genome allowed us to identify putative *SLT* sequences in four different scaffolds of this genome. In particular, the entire sequence of *SaSLTa* matched on scaffold 1182 and 113, whereas the partial *SaSLTb* was found in tandem repeated on both scaffolds 1318 and 554.

In the deduced amino acid sequences of SaSLTa, we identified four TrkA-C domains (Figure S1a), two Anion ArsB/NhaD permease domains (Figure S1a), and a Na:sulfate cotransporter signature containing three amino acids mutated with respect to Prosite pattern PS01271 (Figure S2). The incomplete SaSLTb fragment contains only one TrKA-C domain and one ArsB/NhaD permease domain (Figure S1b), and the tandemly repeated SaSLTb fragments were homologues to the same *Raphidocelis subcapitata* sequence (GenBank: GBF94386.1). Despite the presence of TrkA-C and Anion ArsB/NhaD permease domains in the deduced amino acid sequences (Figure S1) of the respective sequenced fragments, the correct characterization of SaSLTs interpreted from ortholog proteins in other organisms is still uncertain. For these reasons, their expression analysis is not yet possible.

### 2.2. Analysis of Chloroplast Sulfate Transporter Genes

We analysed the previously identified *SaSULP1*, *SaSULP2*, *SaSabc* and *SaSBP* sequence genes, coding for the four subunits of the chloroplast ABC transporter holocomplex responsible for the routing of sulfate into the plastid for reductive assimilation.

#### 2.2.1. SaSULPs

**Figure 2:**
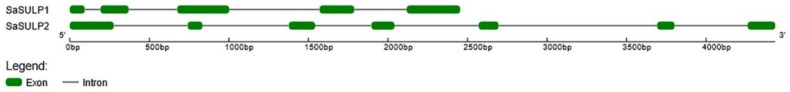
Structures of two *SaSULPs* genes.

*SaSULP1* and *SaSULP2* mapped, respectively, onto scaffold 990 and scaffold 341 of the *T. obliquus* UTEX393 genome. The gene structures are shown in Figure 2.

Sequence analysis of the deduced amino acid sequence of the *SaSULP1* gene, which putatively encodes a membrane-bound protein, showed high homology with *C. reinhardtii* SulP, sharing a 53.9% identity and 64.8% similarity. Analysis of the amino acid sequence of SaSULP1, using the ChloroP tool, indicated the presence of a chloroplast transit peptide consisting of the first 81 amino acids of the precursor protein (Figure S3a). Based on alignment of SaSULP1 and *C. reinhardtii* SulP, in agreement with Lindberg and Melis (2008) [25], we identified six alpha-helix transmembrane domains (Figure S3a).

The *SaSULP2* gene was homologous to *C. reinhardtii SulP2*, with a 67.1% identity and 77.3% similarity in the amino acid sequence and to SaSULP1 (25.5% identity, 43.1% similarity). The SaSULP2 sequence was analyzed as described for SaSULP1, identifying a chloroplast transit peptide (the first 74 amino acids of the precursor protein) and six alpha-helix transmembrane domains (Figure S3b).

#### 2.2.2. SaSabc

**Figure 3:**
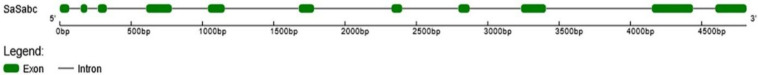
Structure of *SaSabc* gene.

The *SaSabc* sequence mapped onto scaffold 956 of *T. obliquus* UTEX393 genome. Its structure is shown in Figure 3.

The deduced amino acid sequence showed 55.9% of identity and 66.1% of similarity with *C. reinhardtii* Sabc. The analysis through ChloroP identified a 49 amino acid chloroplast transit peptide at the 5′end. Moreover, in agreement with a report by Lindberg and Melis (2008) [25], six conserved motifs, common to the ABC transporter ATP hydrolyzing subunits, were identified through alignment between Sabc and CysA. These motifs are highly conserved between eukaryotic and prokaryotic organism sequences (Figure S4) and are identical to those found in *C. reinhardtii* Sabc (Figure S5). Four of them were involved in binding and hydrolysis of ATP, whereas the remaining two were probably involved in ATP hydrolysis and/or interaction with the membrane-spanning subunits (Figures S4 and S5).

#### 2.2.3. SaSBP

**Figure 4:**
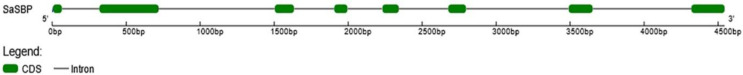
Structures of *SaSBP* gene.

The gene encoding for the Sulfate Binding Protein mapped onto scaffold 1250 of *T. obliquus* UTEX393 genome and its structure is shown in Figure 4.

The deduced amino acid sequence shows 49.5% of identity and 58.8% of similarity with *C. reinhardtii* SBP. Even though gene sequence is incomplete at 5′, the SaSBP conserved part between eukaryotic SBP and prokaryotic SBPA proteins stretches from amino acid 19 to amino acid 325 (Figure S6); no chloroplast transit peptide was identified.

### 2.3. Phylogenesis Analysis of Sulfate Transporter

The phylogenetic analysis of *S. acutus* SLTs, SULTRs and of the chloroplastic ABC transporter subunits, performed through the alignment with homologous sequences retrieved in NCBI data banks, showed similarity and differences in comparison to the most studied *C. reinhardtii* sulfate transport system.

**Figure 5:**
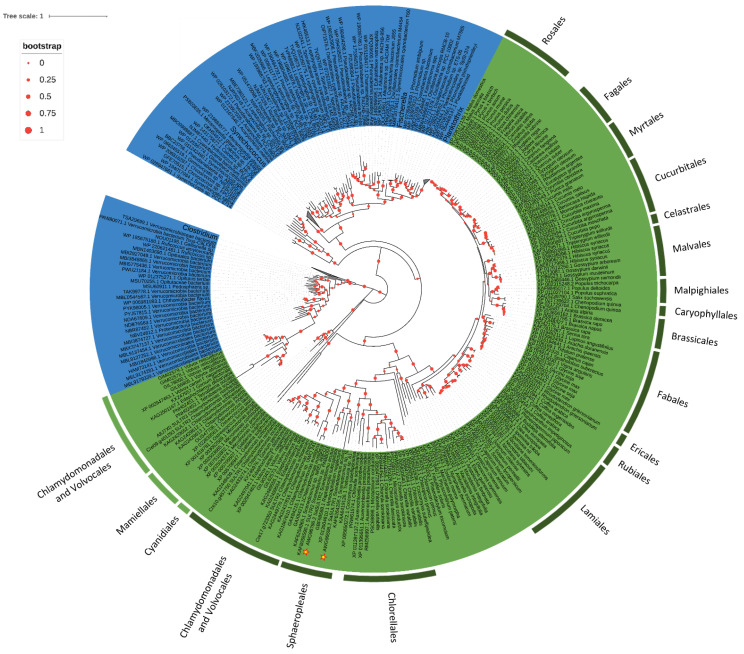
The evolutionary history of SULTRs. The tree with the highest log likelihood (−156,290.01) is shown. The percentage of trees in which the associated taxa clustered together is shown below the branches. The tree is drawn to scale, with branch lengths measured in the number of substitutions per site. This analysis involved 359 amino acid sequences (Table S1) for a total of 2017 positions in the final dataset.

Algal SULTRs transporters are split into two clusters, one including *C. reinhardtii* SULTR3 and the other including *C. reinhardtii* SULTR1 and SULTR2. With some differences in the first residue, these sequences contain the SLC26 signature consensus pattern, STAS domain (Prosite pattern PS50801) (Figure 5). Sequences homologous to SULTR1 and SULTR2 have their procaryotic counterparts in cyanobacteria and are found in the red alga *Galdieria sulphuraria* and in some Bacillariophyceae and, among the Chlorophyta, in the classes Chlorophyceae, Trebouxiophyceae but not in the early diverging group Prasinophyceae (Figure 5). These transporters cluster according to algal taxa (Chlorellales, Trebouxiales, Sphaeropleales, Chlamydomonadales and Volvocales), independently from the transporter type, suggesting that a recent gene duplication, or one subsequent to the separation of the different algal orders, occurred between the two transporters. SULTR1/SULTR2 transporters are closely related to the sulfate transporters belonging to 4.1–4.2 groups of land plants (Figure 5). SULTR3 homologous sequences have been found only in Chlamydomonadales and Volvocales; they cluster in a branch close to bacteria belonging to Verrucomicrobia and Firmicutes. Sequences for SULTR transporters belonging to Mamiellales cluster on a distinct, but close, branch (Figure 5). We did not find a sequence homologous to SULTR3 in *S. acutus*. Moreover, the Clamydomonadales and Volvocales sequences homologous to *C. reinhardtii* SULTR3 contain a Rhodanase domain, located in the 3′portion of the protein, which is not present nor in the bacteria nor in the Mamiellales proteins.

Differences between the SULTR3 and SULTR1-2 algal proteins, were also evidenced in the STAS domain of the relative sequences since they cluster in separated branches even when phylogenetic analysis was conducted only on this domain fragment (not shown).

In *C. reinhardtii* SLT transporters have structures similar to that of SAC1 (Sulfur Acclimation Protein 1), from which they derived their name (SAC1 Like transporters), thus the phylogenetic analysis on the sequence homologous to SaSLTa, was conducted including sequences homologous to *C. reinhardtii* SAC1. SAC1 homologous sequences contain two TrkA-C domains and the Sodium:sulfate symporter family signature Consensus pattern (Prosite Pattern PS01271) (Figure S2b). SAC1 homologous sequences were found in different bacteria taxa that split in different branches, whereas in algae they seem present only in Chlamydomonadales and Volvocales, among Chlorophyta, and in red algae. All these sequences share a true Prosite Pattern PS01271.

SLT homologous sequences are characterized by three to four TrkA-C domains and by a sequence very close to the above mentioned Prosite Pattern with few differences in the strictly conserved amino-acids, depending on the taxa to which they belong (Figure S2b). The changes in the Prosite Pattern involve ambiguous amino acids, with the exception of Chlorellales in which two variations involve the fixed amino acids proline (substituted by serine) and valine (substituted by alanine or leucine), and diatoms in which the *N*-terminal valine is substituted by isoleucine.

**Figure 6:**
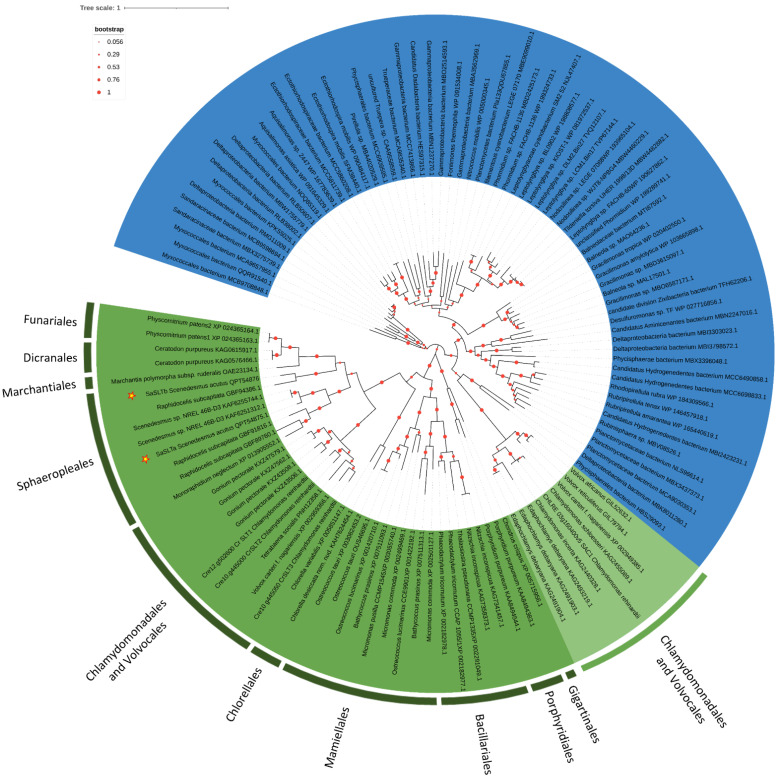
The evolutionary history of SLTs. The tree with the highest log likelihood (−88,409.27) is shown. The percentage of trees in which the associated taxa clustered together is shown below the branches. The tree is drawn to scale, with branch lengths measured in the number of substitutions per site. This analysis involved 117 amino acid sequences (Table S1) for a total of 1769 positions in the final dataset.

SLT transporters were found in diatoms, in the Mamiellales (Prasinophyta), in the Chlorellales, Sphaeropleales, Chlamydomonadales and Volvocales (Chlorophyta), and some sequences were also found in Bryophyta. All algal orders cluster independently, and each genus includes a variable number of sequences. Two SLT genes were found in almost all the Mamiellales, while the phylogenetic tree confirmed the presence of three SLTs transporters in *C. reinhardtii*, SLT3 clustering together with one sequence of *V. carteri* and one of *Tetrabaena socialis* and SLT1 and SLT2 clustering very close on the same branch. Very closely to *C. reinhardtii*, SLT transporters cluster four sequences of *Gonium pectorale*, but their distribution is not sufficiently clear to state if this number corresponds to real different isoforms. Sphaeropleales are closer to Bryophyta, but the number of SLT transporters in the species belonging to this order is far from understood. Two sequences of *Scenedesmus* sp. NREL 46B-D3 cluster close to SaSLTa, whereas only one sequence was retrieved in *Monoraphidium neglectum* and at least four in *R. subcapitata.* Two sequences of this latter species cluster on the same branch as the sequence of *M. neglectum*; one was excluded from the analysis, being partial and too short to be correctly aligned (GenBank: GBF96422.1), and the fourth (GenBank: GBF94386.1) clustered with the partial SaSLTb hypothetical protein on a branch completely separate from the other Sphaeropleales (Figure 6). This last apparently contains only three TrkA-C domains and a PS01271 like sequence similar to that found in Chlamydomonadales/Volvocales. Therefore, this protein seems to have an evolutionary history and/or functional characteristics different from those of the two proteins clustering with *M. neglectum*. From these observations, it may be argued that in the genome of Sphaeropleales, at least two or three SLTs are present.

**Figure 7:**
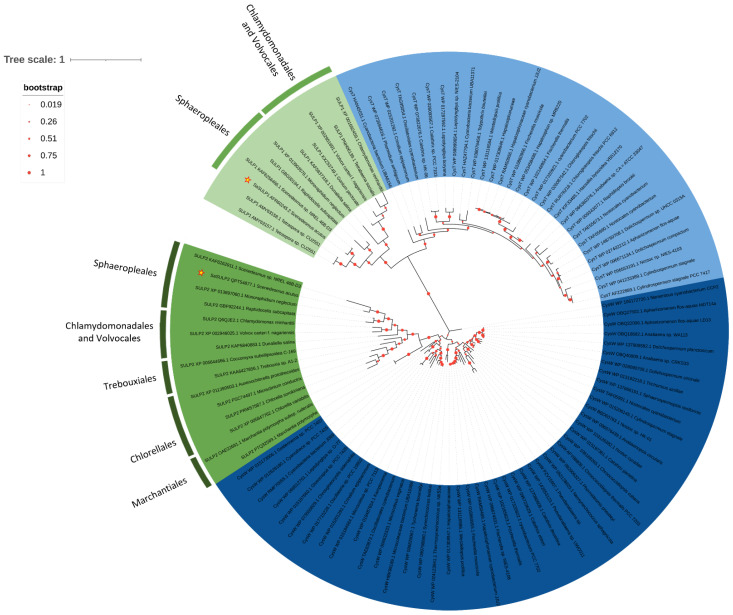
The evolutionary history of SULPs. The tree with the highest log likelihood (−25,726.73) is shown. The percentage of trees in which the associated taxa clustered together is shown below the branches. The tree is drawn to scale, with branch lengths measured in the number of substitutions per site. This analysis involved 103 amino acid sequences (Table S1) for a total of 1036 positions in the final dataset.

As already reported for other green algae, the subunits of the chloroplast ABC transporter cluster with the homologous sequences of prokaryotic ABC sulfate transporter. SaSulP1 and SaSulP2 cluster in two different groups corresponding respectively to CysT and CysW subunits of the prokaryotic holocomplex of the ABC transporter (Figure 7). Nuclear SulP1 sequences retrieved from data bank belonged to Chlamydomonadales, Volvocales and Sphaeropleales and Tetrasporales, while all the Trebouxiophycean sequences were chloroplastic and thus we excluded them from phylogenetic analysis. On the other hand, the SULP2 group includes nuclear sequences from Chlorellales and Trebouxiales and from the Bryophyta *Marchantia polimorpha*.

**Figure 8:**
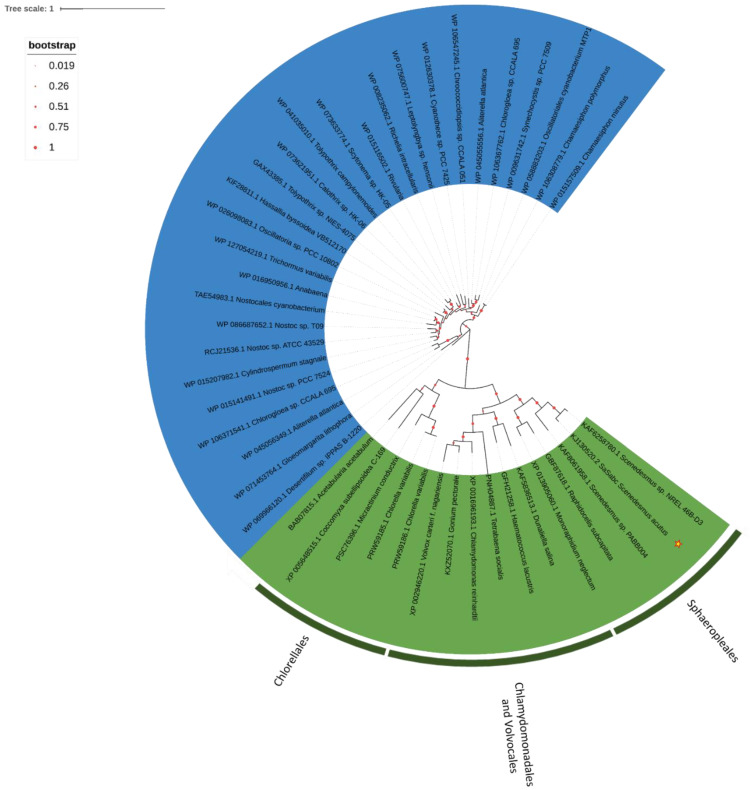
The evolutionary history of Sabc. The tree with the highest log likelihood (−14,456.88) is shown. The percentage of trees in which the associated taxa clustered together is shown below the branches. The tree is drawn to scale, with branch lengths measured in the number of substitutions per site. This analysis involved 44 amino acid sequences (Table S1) for a total of 590 positions in the final dataset.

Similar divergence between eukaryotic and prokaryotic subunits of the sulfur ABC-transporter was observed for Sabc homologous to procaryotic CysA. For this gene, nuclear sequences were found in Chlamydomonadales/Volvocales and Sphaeropleales, which grouped on very close branches, and in the Trebouxiophyceae *Chlorella sorokiniana* and *Coccomyxa subellipsoidea* (Figure 8), while in other Trebouxiophyceae this gene remains chloroplastic.

**Figure 9:**
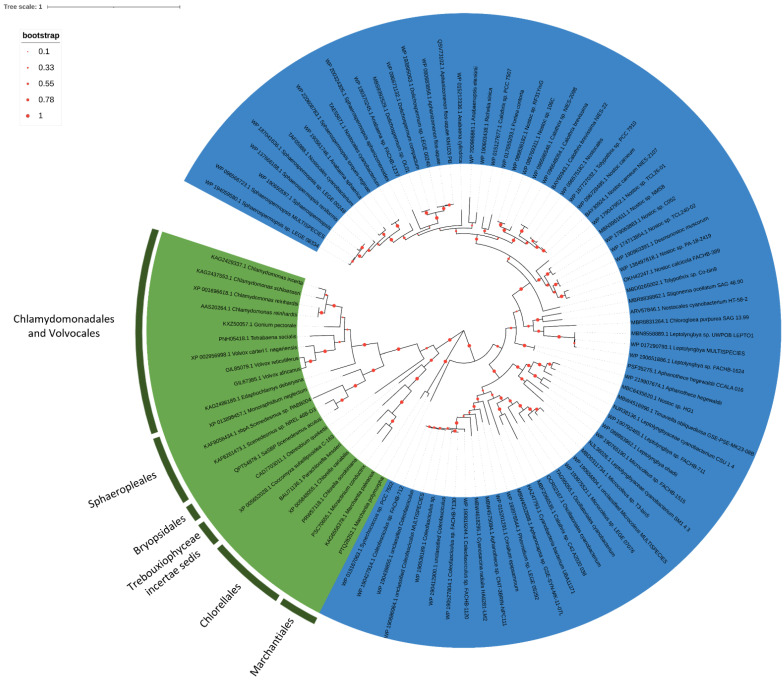
The evolutionary history of SBP. The tree with the highest log likelihood (−30,114.72) is shown. The percentage of trees in which the associated taxa clustered together is shown below the branches. The tree is drawn to scale, with branch lengths measured in the number of substitutions per site. This analysis involved 95 amino acid sequences (Table S1) for a total of 2323 positions in the final dataset.

A distribution on very close branches for Chlamydomonadales/Volvocales and Sphaeropleales separated from Trebouxiophyceae was observed for SBP, homologous to cyanobacteria CysP (Figure 9). The two sequences retrieved for the Bryophyta *Marchantia polymorpha* and *Marchantia paleacea* cluster on an independent branch (Figure 9).

All the subunits of the holocomplex of the algal ABC transporters cluster in different sub-branches following algal taxa.

### 2.4. aqPCR of SULTRs and SULPs Genes

To understand the role of sulfur uptake in Cr-tolerance, and in the transient Cr-tolerance increase induced by S-deprivation, *SULTRs* expression was quantified by aqPCR in both strains after the different treatments.

#### 2.4.1. SULTRs Gene Expression

**Figure 10:**
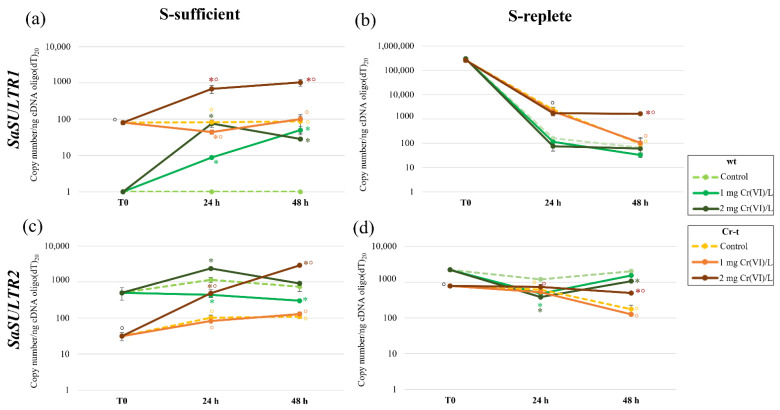
aqPCR of *SaSULTR1* (**a**,**b**) and *SaSULTR2* (**c**,**d**) in both S-sufficient and S-replete condition. Statistical analysis was performed using one-way ANOVA with the Tukey *post hoc* test (*p* < 0.05) after the Shapiro-Wilk normality test. *: significant difference between Cr-treated (with 1 or 2 mg Cr(VI)/L) and untreated cells within strain. °: significant difference between the two strains in similar conditions.

When grown in control S-sufficient conditions (Figure 10a) *SaSULTR1* was expressed only by Cr-t cells, and in both strains its expression level did not change during 48 h of culture. In the wt strain, *SaSULTR1* was significantly induced (*p* < 0.001) by chromium exposure at both the tested concentrations (even with different time courses) and reached the level observed in the Cr-t control. In the Cr tolerant strain, *SaSULTR1* expression dramatically increased (9–13 fold that of the control) exclusively at LOEC (2 mg Cr(VI)/L); whereas, in presence of 1 mg Cr(VI)/L, *SaSULTR1* expression showed an initially low but significant decrease after 24 h of metal exposure, but remained substantially stable within the 48 h treatment (Figure 10a).

S-starvation induced strong *SaSULTR1* expression in that the transcript level was extremely high in both strains after preculture in S deprived medium (Figure 10b). Moreover, in these conditions nutritional stress was clearly predominant, masking chromium effects in S-replete cells during the first 24 h culture. Even in S-replete conditions, however, a different behavior was observed between strains since after nutrient resupply, *SaSULTR1* was downregulated more rapidly in wt than in Cr-t cells. Besides, the Cr-t strain maintained a high level of *SaSULTR1* expression upon 48 h of 2 mg Cr(VI)/L exposure. After 48 h from medium renewal, in S-replete cells of both strains, *SaSULTR1* expression was still significantly higher (*p* < 0.0001) than in S-sufficient cells (Figure 10a,b).

In contrast, we found a higher *SaSULTR2* expression in the wt than in the Cr-t strain at the end of preculture in +S medium (T0) (Figure 10c). The expression of this gene in S-sufficient conditions increased in both strains after 24 h from medium renewal, both in the control and in the 2 mg Cr(VI)/L-exposed cells. By contrast, in the presence of 1 mg Cr(VI)/L, the expression significantly decreased in wt and was not affected at all in the Cr-t strain, where it perfectly overlapped the control time course (Figure 10c).

In S-starved cells, even *SaSULTR2* expression was enhanced, and both strains at the end of pre-culture (T0) in −S medium showed transcript levels higher than in non-starved cells (4.4- and 25-fold in wt and Cr-t, respectively) (Figure 10d).

In wt S-replete cells, *SaSULTR2* expression decreased after 24 h from nutrient resupply, though remaining higher than in the S-sufficient condition, and Cr-exposure induced a further significant decrease. *SaSULTR2* transcription levels were restored thereafter (Figure 10d).

In Cr-t strain S-replete cells, *SaSULTR2* expression remained unchanged at 24 h and decreased to basal level after 48 h from nutrient resupply, with the only exception of 2 mg Cr(VI)/L-exposed cells as already observed for *SaSULTR1* (Figure 10d).

A comparison between *SaSULTR1* and *SaSULTR2* copy number (Table 1) indicated that at the end of preculture in +S medium (T0 in S-sufficient condition) the wt expressed a higher total number of transporters than the Cr-t strain (500 ± 54 vs. 112 ± 7), whereas this latter assured S uptake through *SaSULTR1* activation (81 ± 9) not expressed in the wt. After medium renewal, both strains increased the transporter number essentially by inducing *SaSULTR2* and leaving unchanged *SaSULTR1* transcription (Table 1).

In the S-replete condition, both strains significantly enhanced S-uptake, increasing the total number of transporters as evidenced both by a huge *SaSULTR1*, induction which reached levels not significantly different in the two strains (T0 in S-replete condition), and the simultaneous increase of *SaSULTR2* (4.4-fold and 25-fold in the wt and Cr-t strain, respectively) (Table 1). Despite the greater *SaSULTR2* relative increase observed in the Cr t strain, the copy number of this gene remained significantly higher in the wt than in the Cr-t strain (Table 1). After 24 h from nutrient re-supply the total copy number of *SULTRs* was higher in Cr-t than in wt, due to the slower silencing of both gene expressions in this strain, *SaSULTR1* expression, however, prevailing in the Cr-t and SaSULTR2 in the wt (Table 1). After 48 h, a further decrease of both genes was observed in both strains, notwithstanding the total copy number remaining higher in the wt than in Cr-t strain and more elevated than in S-sufficient condition.

#### 2.4.2. SULPs Gene Expression

To investigate if sulfate intake in the chloroplast is differently regulated in the two strains, we quantified the transcription of the genes encoding the channel of the chloroplast sulfate transporter (*SaSULP1* and *SaSULP2*) under the different growth conditions through aqPCR.

**Figure 11:**
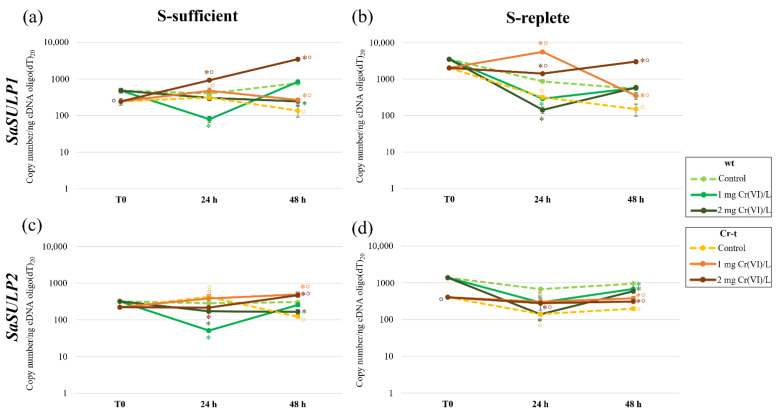
aqPCR of *SaSULP1* (**a**,**b**) and *SaSULP2* (**c**,**d**) in both S-sufficient and S-replete conditions. Statistical analysis was performed by using one-way ANOVA with the Tukey *post hoc* test (*p* < 0.05) after the Shapiro-Wilk normality test. *: significant difference between Cr-treated (with 1 or 2 mg Cr(VI)/L) and untreated cells within strain. °: significant difference between the two strains in similar conditions.

In S-sufficient cells, *SaSULP1* expression was two-fold higher in the wt than in the Cr-t strain, and chromium exposure induced an opposite response in the two strains as a decrease of expression was observed in the wt and an increase in the Cr-t strain (Figure 11a).

As occurred for SULTRs transporters, S-starvation induced a marked accumulation of *SaSULP1* transcript in both strains (7.3-fold in wt and 8-fold in Cr-t) although the wt strain showed significantly higher levels than the Cr-t strain. In the S-replete condition, *SaSULP1* expression decreased more slowly in the wt than in Cr-t strain. Chromium exposure induced a significant decrease in *SaSULP1* expression in the wt after 24 h and initial levels were restored thereafter (Figure 11b). In the Cr-t strain, instead a similar decrease was observed in control cells, while in chromium-exposed cells *SaSULP1* transcription remained higher than in control and a further increase was observed after 24 h at 1 mg Cr(VI) L^−1^ (Figure 11b).

In S-sufficient conditions, the variations observed in *SaSULP2* expression were less than those of *SaSULP1* (Figure 11c). However, for this gene, the transcription decreased in wt cells exposed to chromium (both at 24 and 48 h). Cr-t instead behaved like the wt at 24 h while a significant increase in transcription was observed after 48 h of chromium exposure (albeit lower than that observed for *SaSULP1*) (Figure 11c).

*SaSULP2* expression was strongly enhanced by S-starvation (T0), most of all in the wt, in which a 4.2-fold increase in copy number was observed (Figure 11d). Notwithstanding, *SaSULP2* expression decreased in both strains after medium renewal, and Cr-t rapidly restored the basal levels (S-sufficient). *SaSULP2* transcription remained significantly elevated (above basal levels) in the wt up to 48 h (Figure 11c,d). Even in the S-replete condition, *SaSulP2* expression was negatively affected by Cr(VI) exposure in the wt and positively in the Cr-t strain (Figure 11d).

**Figure 12:**
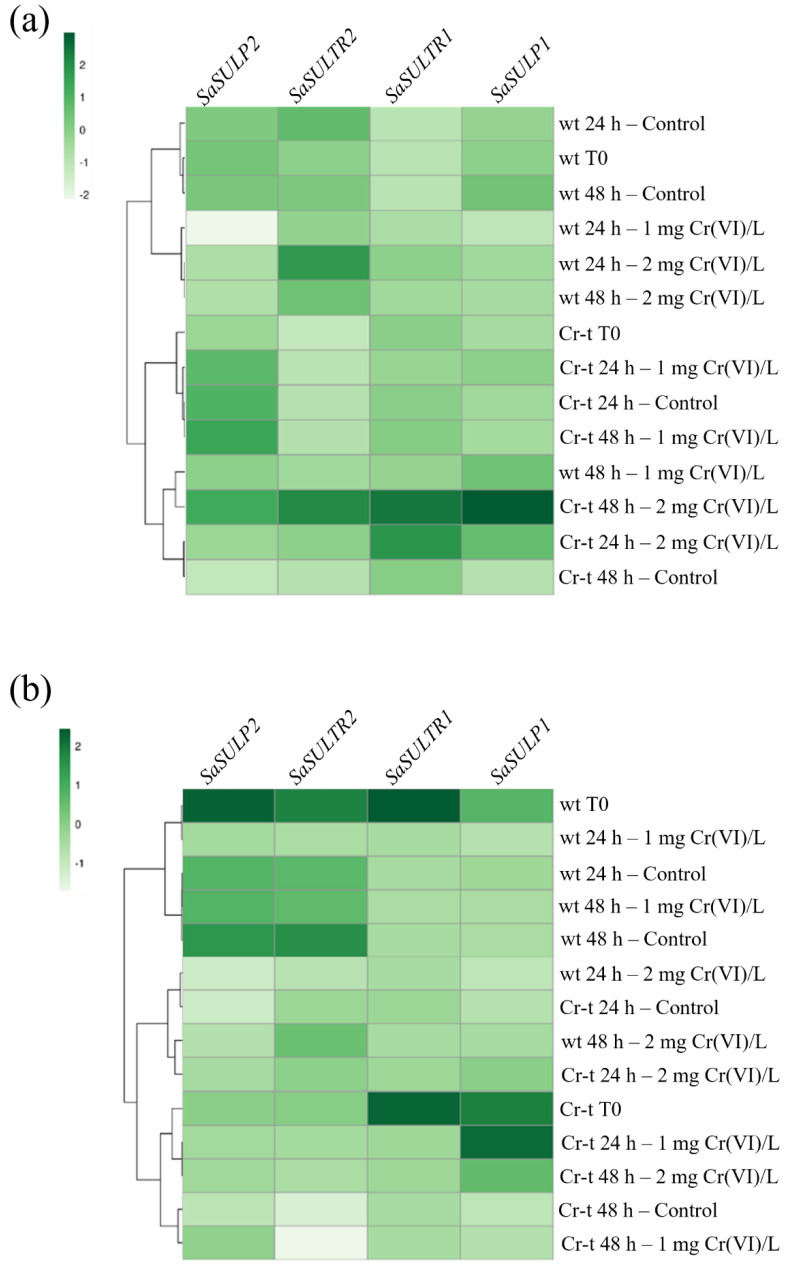
Heatmap of *SaSULTRs* and *SaSULPs* expression in cells growth in S-sufficient (**a**) and S-replete (**b**) condition. Genes with higher expression levels are shown in dark green, whereas genes with lower expression levels are shown in light green.

As shown in Figure 12, a correlation between the expression pattern of *SULTRs* and *SULPs* transporters in the different growth conditions was found in both S-sufficient and S-replete cells. In the S-sufficient condition, we observed a clear division in the two groups discriminating the expression in wt vs. Cr-t strains (Figure 12a). The first group included most of the wt samples, whereas the second one grouped Cr-t samples and wt cells exposed to 1 mg Cr(VI)/L for 48 h (Figure 12a). The wt group was, in turn, divided into two subgroups: the controls and the Cr-exposed samples, regardless of the treatment duration (Figure 12a). The Cr-t group was also subdivided into subgroups but in this case the controls at shorter growth time cluster together with the samples exposed to Cr(VI) beyond their LOEC (2 mg Cr(VI)/L), regardless of treatment duration. The other subcluster included the control at 48 h, all the samples exposed to 2 mg Cr(VI)/L and the wt exposed to its proper LOEC (1 mg Cr(VI)/L) for 48 h (Figure 12a).

In the S-replete condition, the heatmap is clearly subdivided into two groups, but different from those observed in S-sufficient condition (Figure 12b). In this case, the first group included the controls and the 1 mg Cr(VI)/L exposed wt samples. Very intriguingly, the second one grouped all the Cr-t samples and all the wt samples exposed to 2 mg Cr(VI)/L (Figure 12b).

## 3. Discussion

### 3.1. Analysis of Sulfate Transporter Genes

To investigate the role of S on Cr(VI) tolerance we analyzed the sulfate transporter system of *S. acutus.*

In *S. acutus*, the previously identified sequences of H^+^/SO_4_^2−^ transporter type (SULTRs) [31] resulted homologous to Cre10.g457750 (SULTR1) and Cre17.g723350 (SULTR2). According to the literature, in *C. reinhardtii* are present three H^+^/SO_4_^2−^ transporters SULTR1-3 [17]. A protein similar to the *C. reinhardtii* SULTR3 (Cre09.g401293), was not found in *S. acutus*. SULTR3 seems absent in Sphaeropleales, since we did not find a homologous sequence for this gene/protein, either in *S. acutus* or in data bank accession for *Raphidocelis*, *Monoraphidium* and in other *Scenedesmus* strains whose full genome sequencing has been recently obtained [32,33,34].

To date, two sequences putatively codifying for putative SLT transporters were identified in *S. acutus*: *SaSLTa* and the incomplete *SaSLTb* [31]. However, SLTs transporter characterization in *S. acutus* is still unclear.

Moreover, *SaSLTb* mapped on a *T. obliquus* scaffold are apparently present as two similar sequences tandemly arranged, for which the correspondence with one or two proteins is not clear, since they both are homologous to the same *R. subcapitata* protein (GenBank: GBF94386.1). The gene organization seems similar to that found in *C. reinhardtii* genome, in which SLT2 and SLT3 are arranged in tandem in a head-to-tail orientation with a partial overlapping of the 3′-untranslated region of one gene with the 5′-untranslated region and the first exon of the other [23]. These two genes show different sulfate affinity and strongly interfere in reciprocal transcription.

The difficulty we encountered in the identification of these two latter SLT genes, has not made possible the expression analysis of the whole plasma membrane transporters and limited the comprehension of their involvement in maintaining the homeostasis of S uptake.

Regarding the chloroplast sulfate transport, all four genes for the subunit of the holocomplex identified in *S. acutus (SaSULP1*, *SaSULP2*, *SaSabc*, *SaSBP)* showed a higher homology with the respective *C. reinhardtii* amino acid sequences. As reported by Lindberg and Melis (2008) [25], the genes codifying for the subunit of the chloroplastic holocomplex, are differentially distributed between the nuclear and chloroplast genome in different algal groups. In *S. acutus*, all the four genes are located in the nuclear genome, as in *C. reinhardtii*.

### 3.2. Phylogenetic Analysis of Sulfate Transporter Sequences

Phylogenetic analysis indicated a distinct evolutionary origin of the sulfate transporters studied in this work. The sulfate transporter system in Chlorophyceae seems quite heterogeneous with regard to both plasma membrane and chloroplast transporters.

SULTR1-2, close to SULTR transporters of group 4 of land plants, cluster according to isoform within the algal order, thus they likely derived from a duplication that occurred after the divergence of the different algal orders. Similar gene duplication seems not to have occurred in Volvocales. Probably, the members of SULTRs subfamily underwent subsequent duplication and specialization to play different roles in the sulfate transport process. A possible explanation for the presence of unique isoforms of these transporters in *V. carteri* was advanced by Pootakham et al. (2010) [23] who hypothesized that sulfur stored in the extracellular matrix of this species can support sulfur uptake, making superfluous the existence of a second transporter.

Since SULTR3 homologous sequences are close to Verrucomicrobia and Firmicutes, it seems that this gene has been lost in both Trebouxiophyceae and Sphaeropleales or acquired from bacteria with lateral gene transfer only in the common ancestor of Chlamydomonadales and Volvocales after their divergence from the other algal groups. Surprisingly homologous proteins of Chlamydomonadales and Volvocales contain a rhodanase domain absent in the related bacteria sequences. Rhodanase (thiosulfate sulfurtransferase) is an enzyme widespread in living organisms acting as sulfur transferase from sulfur donors to nucleophilic sulfur acceptors [35]. This enzyme can play a role in detoxifying cyanide, ROS and heavy metals, but its function and cell localization is not yet completely understood [35]. Sulfontransferase activity was, however, found in the plasma membrane fraction. It likely confers on SULTR3 protein the capacity to interact with sulfur compounds other than sulfate. In some freshwater bodies, a great proportion of S can be contained in organic molecules [26], and the presence of transporters with a sulfotransferase activity and with arylsulfatase can confer an high environmental adaptability.

SULTR transporters of the early diverging green algae Prasynophyceae cluster on a distinct branch closest to SULTR3 than to SULTR1-2.

Sequences homologous to the *C. reinhardtii* Na^+^/SO_4_^2−^ transporters have been found in Chlorellales, Sphaeropleales, Chlamydomonadales, Volvocales and in the Bryophyta *Phiscomytrium*, *Ceratodon* and *Marchantia*. The number of isoforms that should be expected in different groups is, however, not clear. In Chlamydomonadales/Volvocales three isoforms were found in *C. reinhardtii*, in which the two isoforms SLT1 and SLT2 are very close and separated from SLT3.

Among the Sphaeropleales, seemingly there is one SLT transporter in *M. neglectum* and two in *Scenedesmus_*sp._NREL_46B-D3, whereas at least three homologous proteins (one of them with divergent features) are present in *R. subcapitata*. Therefore, we expect to find more than two genes for these transporters in *S. acutus*. This distribution shows the complex situation that limited our analysis in *S. acutus*.

Na^+^/SO_4_^2−^ transporters are advantageous in oceans where Na^+^ concentrations are high; these transporters are thus likely preferred in sea water. Moreover green algae evolved in Paleozoic, when sulfate concentration in oceans was low, due to the scarcity of oxygen in atmosphere [21]. Today sulfate abundance varies in space and time, and in freshwater environments is lower than in oceans. Moreover, the diminished antropic SO_2_ emission imposed by legislation to limit acid rains problems, caused a reduction in S precipitation from the atmosphere, resulting in sulfate becoming almost limiting in some oligotrophic lakes [26,36]. The retention of both Na^+^/SO_4_^2−^ and H^+^/SO_4_^2−^ transporters, has thus been interpreted as the capacity of algae to survive in different environmental conditions [23].

Sequences similar to CHLRE_03g160400v5 (SAC1), homologous to bacterial and red algae SLT transporters, among green algae were found only in Chlamydomonadales and Volvocales, in which they are hypothetically involved in sulfur perception. Thus, they likely have evolved only in these algal groups and have a different origin from SLT1-3, as in bacterial SLT transporters. Chlamydomonadales/Volvocales SAC1 proteins contain two TrkA-C domains, the true signature of Sodium:sulfate symporter family, while the SLT type transporters seem to have gained additional TrkA-C domains and mutations in the Sodium:sulfate signature. The Prosite pattern for this motive is based on 22 animal sequences and is similar to that found in bacterial SLC13 transporters. The amino acidic changes in this sequence are different and conserved according to algal taxa. It is not excluded that the mutations accumulated in this motive are functional for algal proteins. SAC1-like proteins are close to the sequences of Deltaproteobacteria, a class of bacteria comprising an ecologically and metabolically diverse group best known for dissimilatory sulphate reduction [37].

In the Sphaeropleales, we considered all the four genes codifying for the chloroplast ABC-transporter subunits located in the nuclear genome, as in *C. reinhardtii*. Despite Wakasugi et al. (1997) [38] reporting that SULP1 and Sabc are chloroplastic genes in *C. vulgaris*, we retrieved SULP1 proteins codified by the nuclear genome in other Trebouxiophyceae.

Phylogenetic analysis revealed that SULPs cluster based on the respectively isoform (SULP1 and SULP2) and each of them, as for the remaining holocomplex genes (Sabc, SBP) cluster in different subbranches following algal taxa. Thus, it seems that an ancestral and horizontal transfer occurred in these genes.

Altogether, these data show that sulfur metabolism in Sphaeropleales cannot be regarded as completely similar to that of the well-studied *C. reinhardtii*, nor for the number of involved genes, or for their specialization/activity.

### 3.3. Analysis of SaSULTRs and SaSULPs Transcription

A different behavior of SULTRs transporters was observed in the two strains in both S-sufficient and S-replete conditions.

In the S-sufficient condition, the wt and Cr-t strains adopted different strategies of S uptake: while wt expressed a higher number of *SaSULTR2* copies, the Cr-t strain reached a more efficient S uptake expressing a lower copy number of *SaSULTR1*. Both strains responded to sulfate resupply by increasing the transcription of *SaSULTR2*, the putatively low sulfate transporter, though Cr-t maintained a basis of transcribed *SaSULTR1*.

Both genes are clearly inducible by S-starvation, but *SaSULTR1* transcription definitely overwhelms *SaSULTR2* expression, thus sulfur uptake in S-replete cells is due to the overlapping of the two transporters activities. This different transporter ratio likely allows Cr-t a more efficient S-uptake with the further advantage in sparing energy for transporter construction. It is interesting to note that in S-replete conditions, Cr-t cells reduce both *SaSULTR1* and *SaSULTR2* transcription more slowly than wt cells, indicating a different regulation in the two strains linked to different inner sulfur perception and/or exploitation. Previous work indicated that sulfur uptake in +S medium is similar in the two *S. acutus* strains or even higher in the Cr-t [28,30]. This result was apparently obtained by a Cr-t strain with a lesser number of transporters; therefore, it would seem that *SaSULTR1* and *SaSULTR2*, respectively, encode a high and low affinity transporter.

In the S-sufficient condition, both genes were modulated by chromium exposure, albeit in a different manner in the two strains. At 1 mg Cr(VI)/L, LOEC for this strain, wt slowly induced the transcription of the putatively high affinity transporter SaSULTR1 and reduced the expression of *SaSULTR2*, whereas the Cr-t strain maintained a similar expression level to the control for both transporters. At 2 mg Cr(VI)/L (LOEC for Cr-t strain) both strains induced the transcription of both genes. The wt responded with a moderate increase in *SULTRs* expression at 24 h (two-fold and ten-fold respectively for *SaSULTR2* and *SaSULTR1*) restoring basal levels thereafter. The Cr-t strain induced the transcription of both genes (6- and 8.3-fold respectively for SaSULTR2 and SaSULTR1, at 24 h exposure) which continued to increase even after 48 h, reaching levels 10 and 22-fold those of the control cells.

S-deprivation induced a strong activation of *SaSULTR1* in both strains, and no differences were observed between control and chromium exposed cells, while *SaSULTR2* in this nutritional condition seemed negatively modulated by chromium in the wt and activated at the LOEC concentration in the Cr-t strain, which once again showed a better capacity in enhancing cell defense capacity.

This hypothesis is in agreement with Marieschi et al. (2015) [29] who speculated that in the wt cells, the reduction in chromium uptake after S-deprivation may occur through the induction of high-affinity sulfate transporters, which are presumably constitutively active in the Cr-t strain and can account for the different Cr(VI) sensitivities of the two strains.

The strong *SaSULTR1* activation after preculture in S-deprived medium, and its silencing after medium renewal, is likely related to the transient tolerance increase observed in both strains after a period of S-starvation [28,29,30]. This hypothesis is strengthened by the increase in sulfur (reaching a significantly higher level in the Cr-t than in the wt strain) and the contemporary decrease in chromium uptake observed in both strains following S-starvation [30]. The authors observed that the stoichiometric ratio (S/Cr) in/out underwent a threefold increase in S-replete cells of both strains, suggesting that the higher sulfur accumulation during recovery after starvation was due to the induction of higher affinity sulfate transporters rather than to a simple increase in transporter number. The induction of high affinity sulfate transporters and the increase in sulfate transporter number during acclimation to S-starvation are well documented both in vascular plant and in algae [23,39,40,41,42,43,44,45]. In *C. reinhardtii,* high-affinity and high-capacity sulfate transporters are activated by S-starvation and function with a mechanism based on secondary active transport supported by a proton gradient generated across the plasmalemma [39].

The sum of this information indicates that *SaSULTR1* is expressed to cope with nutritional stress, and that chromium induces a sort of S-starvation effect as suggested by many authors [6,8,46].

Sulfate uptake and assimilation are tightly regulated in a demand-driven manner through a complex network of coordinated responses that involve transcriptional and post-transcriptional mechanisms as well as protein-protein interactions. In *A. thaliana* O-acetylserine (thiol) lyase (OASTL), the enzyme responsible of Cys synthesis interacts with SAT in the formation of the Cysteine Synthase Complex (CSC) and can also affect sulfate uptake and Cys synthesis via direct interaction with the STAS domains of SULTRs transporters [47]. Following S-starvation, the whole pathway is activated [42,44] with positive feedback by the accumulation of OAS [48] and repressed with negative feedback by GSH or Cys [49,50,51].

In *Chlamydomonas* some sulfur availability signaling proteins have been identified, including SAC1, a plasma membrane protein (similar to SO_4_^2−^ transporters) that appears to function as a sensor of SO_4_^2−^ levels. When no SO_4_^2−^ is bound to SAC1, serine-threonine kinase SNRK2.1 is activated and elicits transcriptional activation of many S-deprivation responsive genes. When the environment is S-replete, serine-threonine kinase SNRK2.2 (also known as SAC3) causes complete repression of S-responsive gene expression, possibly by phosphorylating SNRK2.1 and inhibiting its activity. Induction of High-Affinity Sulfate Transporters (HASTs) during S deprivation is SAC1-dependent, although there is some increase in HAST activity in the sac1 mutant that may represent post-translational regulation. Moreover, it is known that SNRK2.2 kinases have an epistatic relationship with SNRK 2.1 kinases, and they have a negative modulation on gene expression of SNRK 2.1 kinases [52]. In higher plants, SNRK2s family members are involved in regulation of plant tolerance to abiotic stresses [53]. Previous studies demonstrated that the SNRK2 family includes ten members in *Arabidopsis* and rice [54]. All of them, except SNRK2.9 in *Arabidopsis*, play a role under different stress conditions such as cadmium, drought, and salinity [53].

Thus, it may be hypothesized there is a similar control mechanism of sulfur uptake pathways in *S. acutus* with a different S perception and/or a disturbed feedback mechanism in the Cr-t strain. However, a protein similar to SAC1 was not found in Sphaeropleales; therefore, to date, the first sensor of sulfate levels in this algal taxon is not known, and it is not possible to determine which kind of control involves *SaSULTRs* induction upon S-starvation

Previous work indicated that the Cr-t strain has a higher Cys level than the wt and a higher GSH level when exposed to metals. It is conceivable, therefore, that positive feedback is differently regulated in the two strains, maybe through a more elevated OAS production/accumulation, as suggested by Sardella et al. (2019) [30]. Emerging evidence suggests that epigenetic regulation of gene expression also plays an important role in the adaptive response to S deficiency and the maintenance of S homeostasis [55]. In our previous work we reported a different methylation level between wt and Cr-t strain as well as after Cr(VI) exposure [56] and demonstrated that hypomethylation of the *SaSULTR1* promoter was linked to its overexpression in the Cr-t strain [31]. These data suggest that epigenetic changes of this gene could be correlated to its differential expression upon Cr(VI) treatment as well as sulfur availability.

Clearly the sulfur uptake picture in *S. acutus* is not complete without the description of SLTs gene expression. SLT transporters catalyze a Na^+^/SO_4_^2−^ antiport [17] and may be responsible for the majority of SO_4_^2−^ uptake when the pH is high and when it is more efficient to use Na^+^ as a counter ion [23]. In *S. acutus*, after 4 days of culture, an increase in pH (ranging between 8.08 and 9.4) was observed, more pronounced in the S-replete than the S-sufficient condition [29]. The involvement of SLTs transporters, especially after basification of the culture medium, can putatively be included in the S-deprivation responses.

In the S-sufficient condition, both *SaSULP* genes were downregulated in the Cr-t strain in comparison with the wt. According to our previous study [31], in standard conditions *SaSabc* appeared under-expressed as a consequence of its hypermethylation in the Cr-t strain.

Globally, in the Cr-t strain, the expression of these genes increased upon Cr(VI)-treatment when, probably, more sulfur was required in the chloroplast to synthesize sulfur-containing molecules and increase the capacity to cope with intracellular chromium, either through chelation and compartmentalization or through an enhanced antioxidant response. Instead, the decreased expression in the wt strain upon Cr(VI) exposure seemed directly linked to the decrease of sulfate uptake. *SaSULPs* transcription seems inversely correlate with Cys production described in the two strains, both in the control and in Cr(VI)-exposed conditions [30], strengthening the idea that the higher Cys amounts observed in the Cr-t strain rely on extra-chloroplastic synthesis, as hypothesized in our previous studies [30,31].

Upon S-deprivation, *SaSULP1* and *SaSULP2* transcription increased significantly in both strains. These results are in agreement with literature data indicating a specific induction of *C. reinhardtii* putative *SulP1* and *SulP2* in response to S-starvation and not upon other nutrient stresses [25,44].

After S-starvation, Cys production was strongly enhanced in both the wt and Cr-t strain [28,30]. In algae, as in plants [57,58,59,60,61,62], the synthesis of Cys can occur in different cell compartments, as demonstrated by the existence of an OASTL cytosolic isoform inducible by S-starvation in *Chlorella sorokiniana* [63]. Sulfate reduction in green algae is, however, apparently localized exclusively in chloroplasts, since the first enzymes of the assimilation reductive pathway, ATP sulfurylases, are located in this cell compartment [64,65]. Once SO_4_^2−^ enters the cell, it must be routed into plastids for reductive assimilation through the ABC-type holocomplex localized in the chloroplast envelope.

Clustering analysis based on *SaSULTRs* and *SaSULPs* expression pattern in the different growing conditions highlights distinct mechanism for coping with chromium stress based on a dissimilar sulfur requirement in the two strains. In both S-sufficient and S-replete conditions, the wt strain was clearly separated from the Cr-t strain. Moreover, the Cr(VI)-treated samples generated a sub-cluster separate from controls, regardless of the treatment duration. This means that the two strains had a different response to S uptake and assimilation in response to Cr exposure. Interestingly, in the S-sufficient condition, the wt strain exposed to 1 mg Cr(VI)/L for 48 h clustered in the Cr-t group since the wt cells upon this treatment showed an expression trend similar, although lower, to that observed in Cr-t cells.

In a specular way, in the S-replete condition, upon 2 mg Cr(VI) exposure the wt samples clustered together with the Cr-t samples. Probably this happened because in S-replete conditions both strains “shift up” their LOEC and the wt assume a behavior more similar to Cr-t [29], expressing S uptake/assimilation-related genes in the same way as Cr-t cells.

In conclusion, the basal expression of different sulfate transporters is likely the basis for the different chromium tolerance in the two *S. acutus* strains, which apparently cope with chromium stress through different strategies linked to a change in sulfate uptake. A period of sulfur starvation induces, in the wt strain, activation of *SaSULTR1*, a sulfate transporter constitutively active in the Cr-t strain. The two strains have, apparently, a “preference” for the two transporters, the wt prevalently modulating *SaSULTR2* expression in standard condition and resorting to *SaSULTR1* only when facing stresses, whereas Cr-t preferentially modulates *SaSULTR1* expression, even in standard conditions. This suggests that the expression of this gene gives an advantage to the Cr-t strain during its selection on chromium-supplemented medium, since it is known that chromate induces a sort of sulfur starvation in the cells.

## 4. Materials and Methods

### 4.1. Analysis of Gene Sequences Related to Sulfate Uptake and Chloroplast Intake

In this work we analyzed and characterized previously identified genes [31] coding for H^+^/sulfate transporter 1 (*SaSULTR1*; GenBank: MG969380.2), H^+^/sulfate transporter 2 (*SaSULTR2*; GenBank: MF457897.2), sodium sulfate co-transporter (*SaSLTa*; GenBank: MT611469.1), sodium sulfate co-transporter (*SaSLTb*; GenBank: MT611470.1), chloroplast sulfate permease SULP1 (*SaSULP1*; GenBank: JN903531.2), sulfate permease SULP2 (*SaSULP2*; GenBank: MT611471.1), chloroplast sulfate transporter (*SaSabc*, GenBank: KJ130520.2) and chloroplast sulfate-binding protein (*SaSBP*; GenBank: MT611472.1).

Sequence homology analyses were performed using BlastX and Blastp (www.ncbi.nlm.nih.gov/BLAST/, accessed on 15 October 2021), ClustalOmega (www.ebi.ac.uk/Tools/msa/clustalo/, accessed on 15 October 2021) and ClustalX (www.clustal.org/clustal2/, accessed on 15 October 2021) and WebLogo [66,67]. Nucleotide sequences were translated into amino acid sequences using the TranslateTool program (web.expasy.org/translate/, accessed on 15 October 2021). The presence of chloroplast transit peptides (cTP) in protein sequences of SaSULP1, SaSULP2, SaSABC and SaSBP and the location of potential cTP cleavage sites were predicted using the ChloroP 1.1 Server [68].

#### Phylogenetic Analysis

Evolutionary history was inferred using the Maximum Likelihood method and the JTT matrix-based model [69] and conducted using MEGA X software [70]. Initial tree(s) for the heuristic search were obtained automatically by applying Neighbor-Join and BioNJ algorithms to a matrix of pairwise distances estimated using the JTT model, and then selecting the topology with superior log likelihood value. The percentage of trees in which the associated taxa clustered together was calculated through the bootstrap test using 1000 replicates.

SULTR1-2-3, SLT1-3 and SAC1 sequences for phylogenetic analysis were searched against *C. reinhardtii* SULTR1-2-3 (available on Phytozome 13 (https://phytozome-next.jgi.doe.gov/, accessed on 20 October 2021)) in plants, algae and prokaryotic NCBI databases. The GenBank accessions of sequences used for the analysis are reported in Table S1. The presence of STAS domains and the SLC26 consensus pattern in these candidate sequences were checked with PROSITE (https://prosite.expasy.org/scanprosite/, accessed on 21 October 2021) with the objective of including sequences of true sulfate transporters, given many analyzed genomes have not been completely annotated.

Considering the chloroplast transporter (SULP1-2, SABC and SBP), only nuclear sequences coding for plastid subunits were included in the phylogenetic analysis.

### 4.2. In Vitro Culture of Scenedesmus Acutus

Two strains of the freshwater green alga *S. acutus* (M.), wild-type (wt) and chromium tolerant (Cr-t), were used as experimental material. The Cr-t strain was isolated by treating the wt population with a sublethal concentration (1 mg/L) of hexavalent chromium (Cr(VI)) for 3 months [71].

Synchronized axenic cultures of the wt and Cr-t strains of *S. acutus* were grown, as described by Marieschi et al. (2015) [29], in US EPA (1978) liquid culture medium at pH = 7.2 ± 0.1, modified by dissolving both micro and macronutrients in distilled water to obtain a final concentration double of that indicated. No organic matter was present in the medium at the beginning of the culture. The algae were maintained in a climate-controlled chamber (23 ± 1 °C) at 16:8 h light/dark regime with 230 µmol m^−2^ s^−1^ light intensity irradiance. The cultures were continuously aerated (sterile filtered air). To perform the experiments with algae in the exponential growth phase, culture medium was renewed 3 days before starting each experiment by adding 1800 mL of fresh medium to 200 mL of algal suspension in 2 L Erlenmeyer flasks (Stock Culture).

All materials and culture media were previously autoclaved for 15 min at 121 °C and 1 atm.

### 4.3. Sulfur Starvation and Chromium Treatments

Aliquots of the stock cultures in exponential growth phase were centrifuged for 10 min at 2200× *g* and washed with distilled water. The pellets were resuspended at 3 × 10^6^ cells/mL density in 2 L of standard culture medium (+S) containing MgSO_4_ (14.36 mg/L), or in sulfate-deprived medium (−S). Since MgSO_4_ is the only source of sulfur in the standard medium, the amount of MgCl_2_ was simultaneously increased to restore standard magnesium concentration in the −S medium. After a 3-day preculture (called T0) in +S and in −S medium, cells of both strains were washed and collected by centrifugation. Subsequently, the cell-containing pellet was resuspended in new liquid culture medium in chromium free conditions (controls) and in medium supplemented with 1 and 2 mg Cr(VI)/L, supplied as potassium dichromate (K_2_Cr_2_O_7_) for 24 and 48 h. The selected concentrations of Cr(VI) represent the LOEC (Lowest Observed Effect Concentration) inhibiting growth, as established in previous experiments [27,28] for the wt and the Cr-t strain, respectively. Treatments and controls were conducted in triplicates. According to previous studies [28,71,72] an initial cell density of 3 × 10^6^ cells/mL was employed to compare the different culture conditions.

### 4.4. Total RNA Extraction and cDNA Synthesis

Culture aliquots from various treatments, and at each experimental time of both strains, were collected by centrifugation, twice washed with double distilled water, frozen in liquid nitrogen, lyophilized, mortar ground in liquid nitrogen and stored at −80° C before RNA extraction. Total RNA was extracted by using the combination of CTAB extraction followed by RNeasy Plant Mini Kit (QIAGEN, Crawley, UK). About 50 mg of cell powder were transferred into 2 mL tube and 1 mL of extraction buffer (2% hexadecyltrimethylammonium bromide (CTAB, *w*/*v*), 100 mM Tris-HCl pH 8, 1.4 M NaCl, 20 mM EDTA; 2% β-Mercaptoethanol) was added. Following an incubation for 40 min at 58 °C, the tubes were centrifugated for 10 min (3000× *g*). After, the supernatant was collected in a clear tube and one volume of chloroform/isoamilic alcohol (24:1 *v*/*v*) was added. After centrifugation for 15 min at 9500× *g*, aqueous phase was transferred into QIAshredder spin columns provided in the QIAGEN kit in accordance with the manufacturer’s protocol. RNA was quantified by Nanodrop (ND-1000), and integrity was checked by an Agilent 2100 Bioanalyzer (Agilent Technologies, Santa Clara, CA, USA). Only RNA samples with an RNA integrity number ≥ 7 were used for cDNA synthesis. The cDNA synthesis was performed by SuperScript™ III using oligo-dT primers (Invitrogen, Carlsbad, CA, USA) from 1 µg of total RNA, according to the manufacturer’s instructions.

### 4.5. Absolute Quantification Real-Time PCR

Expression of selected genes was evaluated by absolute quantitative real-time PCR analysis (aqPCR). The primers of *SaSULTR1* were designed according to Ferrari et al. (2020) [31]. The primers for *SaSULTR2*, *SaSULP1* and *SaSULP2* were designed using Primer Express™ Software v3.0.1 (Applied Biosystems, Foster City, CA, USA) and are reported in Table 2.

Primer pairs were selected according to their robustness, specificity and consistency.

A calibration curve was generated by using serially diluted standards of known concentrations and produced a linear relationship between Ct and the logarithm of the initial amount of total template DNA [73]. The DNA fragments, used to generate the calibration curves, were produced by PCR [74] with the same primers used for subsequent quantification (Table 2). The single amplified PCR product was verified based on size in a 3% agarose gel under UV illumination. The gel band containing the DNA target was excised and purified by a StrataPrep DNA Gel Extraction Kit (Agilent Technologies, Santa Clara, CA, USA) to recover the amplified product. The concentration of the amplified product was quantified using a Qubit 2.0 Fluorometer (Invitrogen, Carlsbad, CA, USA). The dsDNA standards were serially diluted to obtain a standard series (differing by 10-fold) from 10^9^ to 10^1^ copy per μL.

Initial copy number for generating calibration curve was calculated using the Equation (1) [75]:

(1) $$Copy\;number=\frac{6.022\;\times \;10^{23}\left(\frac{copy}{mol}\right)\times dsDNA\;amount\;\left(g\right)}{dsDNA\;length\;\left(bp\right)\times 650\times 10^{9}\left(\frac{\frac{g}{mol}}{bp}\right)}$$

Amplification reactions were performed according to Ferrari et al. (2020) using the Select SYBR^®^ Green PCR Master Mix (Applied Biosystems, Foster City, CA, USA) in a STEP ONE instrument (Applied Biosystems, Foster City, CA, USA). Target gene copy number was determined by reading the standard series with the Ct values of each sample and reported as the number of molecules per nanogram cDNA. Statistical analysis was performed using one-way ANOVA with the Tukey *post hoc* test (*p* < 0.05) after the Shapiro-Wilk normality test and Levene’s homoscedasticity test using SPSS 25 software (IBM Corporation, Armonk, NY, USA) (http://www-03.ibm.com/software/products/it/spss-statsstandard, accessed on 15 October 2021). The heat maps for correlation between *SaSULTRs* and *SaSULPs* gene expression and growth condition were created by the ClusVis web tool [76].

## Figures and Tables

**Table 1:** Copy number/ng cDNA oligo(dT)_20_ encoding SaSULTR2 and SaSULTR1 transporter in untreated cells in T0 and after 24 and 48 h from medium renewal. Statistical analysis was performed within the column by using one-way ANOVA with the Tukey *post hoc* test after the Shapiro-Wilk normality test. Data are reported as mean value ± standard deviation. Different letters label significant different values (*p* < 0.05).

Condition		Strain	Copy Number/ng cDNA Oligo(dT)_20_		
			*SaSULTR1*	*SaSULTR2*	SUM of *SaSULTR1* and *SaSULTR2*
S-sufficient	T0	wt	0 ± 0 ^e^	500 ± 54 ^e^	500 ± 54 ^h^
		Cr-t	81 ± 9 ^cd^	31 ± 8 ^h^	112 ± 7 ^g^
	24 h	wt	0 ± 0 ^e^	1150 ± 64 ^bc^	1150 ± 64 ^de^
		Cr-t	82 ±13 ^cd^	102 ± 18 ^g^	184 ± 28 ^g^
	48 h	wt	0 ± 0 ^e^	737 ± 98 ^de^	737 ± 98 ^ef^
		Cr-t	87 ± 32 ^cd^	109 ± 11 ^g^	196 ± 87 ^g^
S-replete	T0	wt	294,000 ± 2777 ^a^	2210 ± 60 ^a^	296,200 ± 2834 ^a^
		Cr-t	256,000 ± 39,939 ^a^	780 ± 72 ^cd^	256,780 ± 39,981 ^a^
	24 h	wt	161 ± 14 ^c^	1195 ± 17 ^b^	1356 ± 27 ^cd^
		Cr-t	2409 ± 614 ^b^	619 ± 71 ^de^	3029 ± 680 ^b^
	48 h	wt	65 ± 20 ^d^	2011 ± 36 ^a^	2077 ± 55 ^bc^
		Cr-t	94 ± 49 ^cd^	174 ± 49 ^f^	268 ± 87 ^g^

**Table 2:** List of primers used for aqPCR of studied genes encoding sulfur transporters.

Gene	Sequence		Efficiency (%)
*SaSULTR1*	FW	5′-TGGCTACCCTTCCAGTATGTTG-3′	94.8
	BW	5′-GGACGTGGACTCAAGCATGT-3′	
*SaSULTR2*	FW	5′-AAGGTGATCCAGGTGGCATT-3′	90.2
	BW	5′-CGCCCCGCTGGTGAA-3′	
*SaSULP1*	FW	5′-CGAGTTTGGCAGCATTGTCA-3′	97.7
	BW	5′-ACTGCTCAAGGCACTGGAAGA-3′	
*SaSULP2*	FW	5′-GCCAACCCTCTGCAGGTATT-3′	90.9
	BW	5′-TTGGTCAGGATGACGCCATA-3′	

## Data Availability

Data is contained within the article and Supplementary Material.

## Supplementary Materials

The following are available online at www.mdpi.com/article/10.3390/plants11020223/s1. Figure S1: SaSLTs amino acid sequences and conserved motifs; Figure S2: Weblogo and Consensus patterns of Sodium:sulfate symporter family signature prosite pattern PS01271; Figure S3: Alignment between SaSULPs and *C. reinhardtii* SulPs amino acid sequences: conserved motifs and transit peptide; Figure S4: Weblogo of Sabc and CysA amino acid sequences; Figure S5: Alignment between SaSabc and *C. reinhardtii* Sabc amino acid sequences: conserved motifs; Figure S6: Alignment between SaSBP and *C. reinhardtii* SBP amino acid sequences: conserved motifs; Figure S7: Weblogo of SBP and CysA amino acid sequences. Table S1: Sequence used to phylogenetic analyses.
