# Supplementary material for: Role of Sulfate Transporters in Chromium Tolerance in Scenedesmus acutus M. (Sphaeropleales)
Source: Plants (Basel). 2022 Jan 15;11(2):223. doi: 10.3390/plants11020223 (PMC8780407; doi:10.3390/plants11020223)
Supplement: Supplementary file 1 [file plants-11-00223-s001.zip › Supplementary Figures.pdf]

(a)

SaSLTa:
MAVAAVALGWQGITVACTLAASLIVMGFDLVGPDLVFGGLTALYVTSGIISIRDGAAGFANTGVLTVLVL
YLVAEGVSQTGGLDLAMNFMLGRASTVFWAQVRMMIPVMVASAFLNNTPICALMIPILISWGRRCGISPK
KLLIPMSFATVLGGTVTLIGTSTNLVVSGLQQEKYGTTDPSKVFQFFTITPYGLPYAIWGMAYIILFS**kw**
Llpgedaaddlnygllvprtsplvgrtakaaglaggkltitgiskghrtpavpytpdmiihegdtlfvtg
svtaveqsvksfalvLLTSDDDVARKSTPGAAVFGAAAADVE**egfdiaasegttnllqvnllkgsqlvgq**
svrqigfrgrfgaaviavkrskalqpgrigdivlqandvlllstgalfdastedftknFRG**lmyldeala**
rqfttavrvgkrskeagktiaevglrginglflfeieradgsllkavdhdtvlamgdvlwfagdldgvay
lqkyttlEHMQA**dqvaklpsdiiyrrlvqvvvshhdlvgkklkevrfrhtygaavlglhrsgqpvagnis**
evplkagdvlvveagpefatnfknnrafSLISEVPNSSPMKRSKMWIALALTVAMVLTQIIGGAIDKEVI
HLWPCAMLTAGAMLAFKCLSADQARESIEWEVYICIAFAFAVSTAMEKTKLALAIANVFVALAQAIGGQT
AALACIYLVTALLSELLTNNAAAAIMYPIASAAAEKLLINPNIMSVAVMLGGSAGWILPYSYQCNLMVYA
AGKYRTKDFVKIGTPYHVWLFVGVVLILGSGDRWQIPVIASLVFTGLVILLPAAYEYLLNDSQKLAVDKK
LHALGSSFRRKRSSNSGEEALVEVYGNGSSKDVSDAANNGAYNGVVAPASLGHRNRSSAGGVA

(b)

SaSLTb:
FTTDLDGVKYLLKDPRLEATQAADAHKLKGRQIYRH**lvqatvspdsalightvremrfrtyyegvvlaih**
rqsgyvslrdvcdvelragdvllleadqsFKKRFKANPAFGLVADVPRSAPLKTRLMWPALGLTAAMVAT
QIISGFTGTSYIDLWPAAILTAAAMLLIGCMTSDQAVEAIDWTVYITIAFAFGVSSAMERSKVAAAIASI
FVKISHAIGGRTAALGSMYLVTGLLSEVLTNNAAAALMYPIAANVGDDMGIQPKLMSVAVMLGASAAFIS
PFGYQCNLMVFTAGDYKTMDFVRLGVLLQVWQLVAACTIFSLPSWWMILCAISLAVVALAVTYAVASSLL
AKRRLKQLKAAASSAGK

Figure S1. SaSLTs amino acid sequences and conserved motifs. Predicted TrkA-C domains were highlighted in lowercase italic and bold font, Anion ArsB/NhaD permease domains were highlighted in underlined font, whereas the Prosite pattern PS01271 highlighted in yellow in both SaSLTa (**a**) and SaSLTb (**b**),

(a)

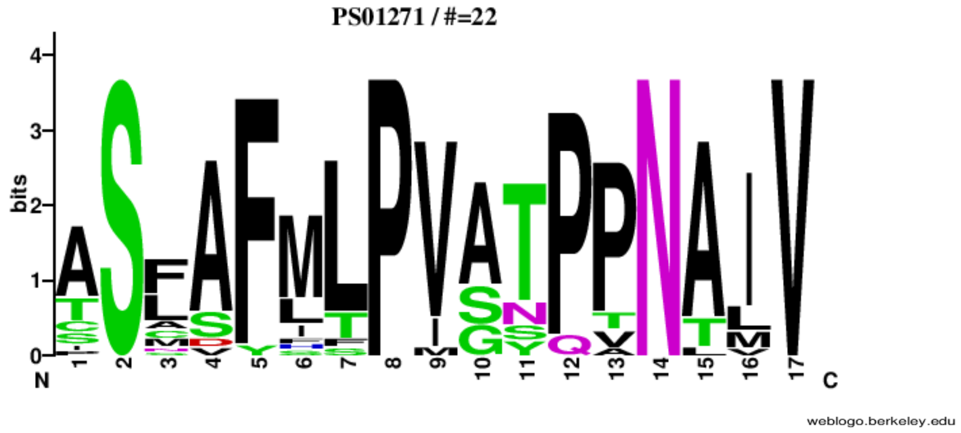

(b)

Bacteria and algal SAC1 proteins

[STACPI]-S-x(2)-[FY]-x(2)-P-[LIVM]-[GSA]-x(3)-N-x-[LIVM]-V

SLT Proteins:

Chlorellales:

[STACPI]-S-x(2)-[FY]-x(2)-S-[FLIVM]-[GSA]-x(3)-N-x-[LIVM]-[VAL]

Chlamydomonadales/Volvocales:

[STACPI]-S-x(2)-[FY]-x(2)-P-[FLIVM]-[GSA]-x(3)-N-x-[LIVM]-V

Sphaeropleales:

[GSTACPI]-S-x(2)-[WFY]-x(2)-P-[YLIVM]-[GSA]-x(3)-N-x-[LIVM]-V

Bryophyta:

[STACPI]-S-x(2)-[FY]-x(2)-P-[YLIVM]-[GSA]-x(3)-N-x-[LIVM]-V

Mamiellales:

[STACPI]-S-x(2)-[FY]-x(2)-P-[FLIVM]-[GSA]-x(3)-N-x-[LIVM]-V

Diatoms:

[STACPI]-S-x(2)-[FY]-x(2)-P-[FYLIVM]-[GSA]-x(3)-N-x-[LIVM]-[VI]

Figure S2. Weblogo (**a**) and Consensus pattern (**b**) of Sodium:sulfate symporter family signature prosite pattern PS01271. In red were highlight the differences between the sequences belonging to the various algal taxa.

(a)

```
SaSULP1      -------------------------mglgaasaaapavsraahrrlvpaaglqqhagvcg   35
CrSulP       mervcshqlassrgrpciagvqrspirlgtssvahvqvspaglgryq-----rqrlqvvA   55
                                      : **::*.*   ** *.   *       :*:  * .

SaSULP1      caaaaaaaagrhirpvpvalqplqasqlqpsrlnppqhqrqqlqt-RLQAAGA-------   87
CrSulP       SAAAAA-----AFDPPG----GVSAGFSQPQQQLPQQHPRQPQAVAEVAVAESVSAPASA   106
             .*****      :  *        :.*.  **.:  * ** **    . .:  .* :

SaSULP1      ----------LSAGGSSSEQPWQPPSSSGK--GFRLPRIQLWDLGGPLAWAYMLGYLAVM   135
CrSulP       APSNDGSPTASMDGGPSSGLSAVPAAATATDLFSAAARLRLPNLSPIITWTFMLSYMAFM   166
                          ** **     * ::::..       *::* :*.  ::*::**.*:*.*

SaSULP1      LILPISALLAKSSLVPLEQFIARATEPVALSAYYVSFSMAIVAGVINAVFGFLLAWVLVK   195
CrSulP       LIMPITALLQKASLVPLNVFIARATEPVAMHAYYVTFSCSLIAAAINCVFGFVLAWVLVR   226
             **:**:*** *:*****: **********: ****:** :::*..**.****:******:

SaSULP1      YEFPGKKWIDAAVDLPFALPTSVAGLTLATVYSEEGVLGSLLMKLGVNVVYTRLGVAVAM   255
CrSulP       YNFAGKKILDAAVDLPFALPTSVAGLTLATVYGDEFFIGQFLQAQGVQVVFTRLGVVIAM   286
             *:* *** :**********************.:*  .:*.:*    **:**:*****.:**

SaSULP1      VFVSFPFVVRTMQPVLQEMEKEVEEAAWSLGASPWTTFTQVLLPPLLPPLLTGTALAFSR   315
CrSulP       IFVSFPFVVRTMQPVMQEIQKEMEEAAWSLGASQWRTFTDVVLPPLLPALLTGTALAFSR   346
             :*************:**::**:********** * ***:*:****** **********

SaSULP1      ALGEFGSIVIVSSNFPFKDLIAPVLIFQCLEQYDFVGATVIGTVLLLISLVMMVGVNWLQ   375
CrSulP       ALGEFGSIVIVSSNFAFKDLIAPVLIFQCLEQYDYVGATVIGTVLLLISLVMMLAVNQLQ   406
             *************** ******************:*****************:.** **

SaSULP1      SYAQRFRK   383
CrSulP       KLARK---   411
             . *::
```

(b)

```
SaSULP2      mealssssllqrr---------asgliastrpclccqhtvsspsslrrn---mesrstqr   48
CrSULP2      ---masttllqpalglpsrvgprsplslpkiprvcthtsapstskycdsssvies-tlgr   56
                ::*::***              * *    . * :* : :. * *.    .    :** :  *

SaSULP2      vqpaaasrravqlqspsnsrrrlvirASGPAGGMGAHGGGRGEPVDTWQKKTILGVAITY   108
CrSULP2      qtsvagrpwlaprpapqqsrgdllvsKSGAAGGMGAHGGGLGEPVDNWIKKLLVGVAAAY   116
                .*.     .    :*.:**  *::   ** ********** *****.* ** ::*** :*

SaSULP2      ILLTVILPFINVFIQAFAKGLGPFLEAVMEHDFIHATKLTLMLAAITVPLNTLFGTVAAI   168
CrSULP2      IGLVVLVPFLNVFVQAFAKGIIPFLEHCADPDFLHALKMTLMLAFVTVPLNTVFGTVAAI   176
             * *.*::**:***:******: ****    : **:** *:***** :******:*******

SaSULP2      LITRNEFPGKVLLLSLLDLPFSISPVVTGLMLMLLYGRAGWFATALADGGLKVVFAFSGM   228
CrSULP2      NLTRNEFPGKVFLMSLLDLPFSISPVVTGLMLTLLYGRTGWFAALLRETGINVVFAFTGM   236
              :*********:*:****************** *****:****: *  : *::*****:**

SaSULP2      LLATLFVTLPFIVRELIPTLEQMDLAQEEAARSLGANPLQVFWHVTLPNIRWGLLYGVIL   288
CrSULP2      ALATMFVTLPFVVRELIPILENMDLSQEEAARTLGANDWQVFWNVTLPNIRWGLLYGVIL   296
              ***:******:****** **:***:******:****  ****:***************

SaSULP2      TNARAMGEFGAVSVISGNIIGRTQTLTLFVESAYKEYNSEAAFAAAVLLSCLALGTLWVK   348
CrSULP2      CNARAMGEFGAVSVISGNIIGRTQTLTLFVESAYKEYNTEAAFAAAVLLSALALGTLWIK   356
              *************************************:***********.*******:*

SaSULP2      DKVEQAAAAAEAAK      361
CrSULP2      DKVEEAAAAAESRK      369
             ****:*****: *
```

Figure S3. Alignment between SaSULPs and *C. reinhardtii* SulPs amino acid sequences: conserved motifs and transit peptide. According to Lindberg and Melis (2008) [25], in both SaSULP1 (a) and SaSULP2 (a), in **lowercase italic and bold font** were highlighted the predicted chloroplast transit peptide; in **red font** the transmembrane domains; in **green font** the regions of protein lie in the chloroplast stroma; **yellow**: regions of protein lie in the intermembrane space.

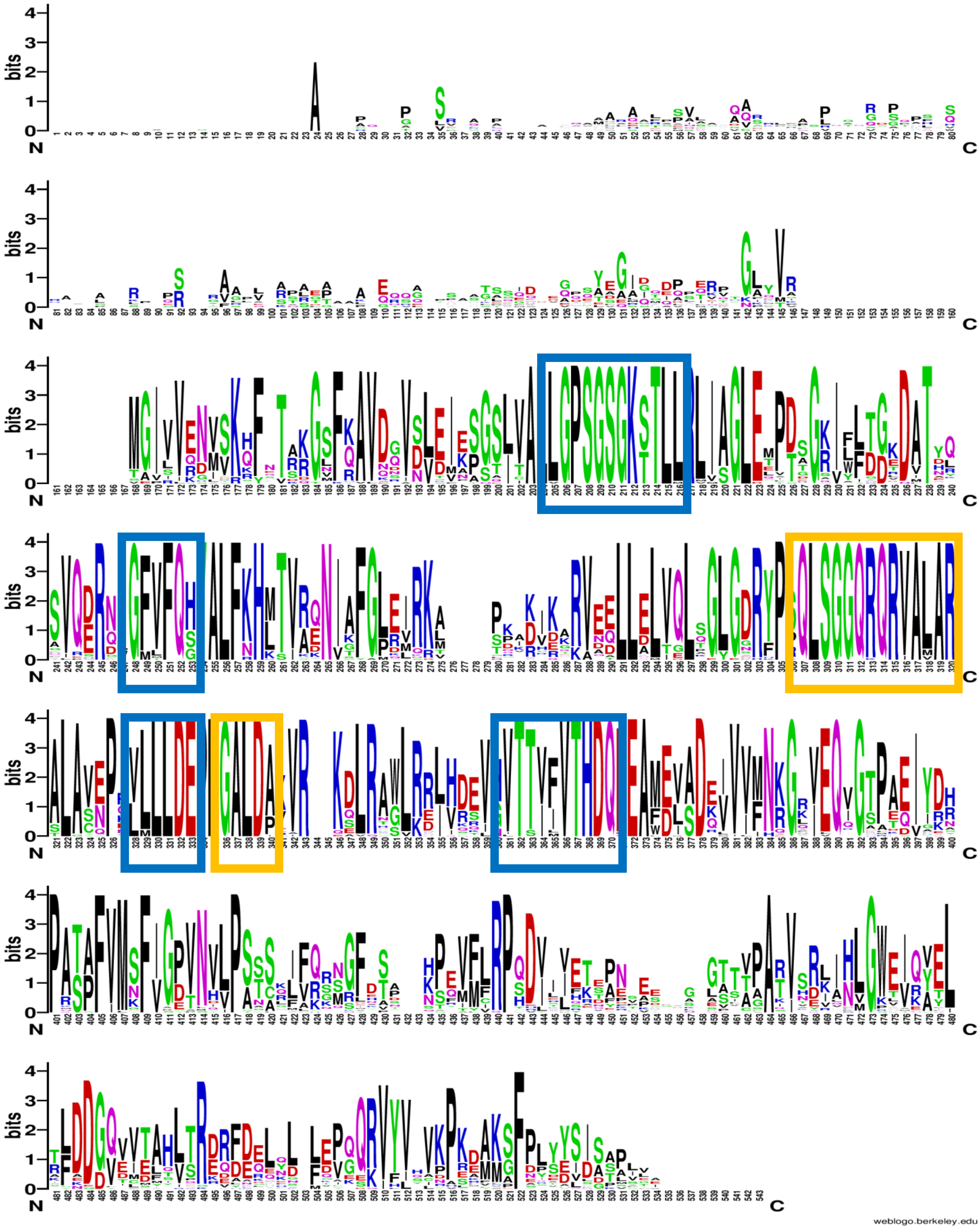

Figure S4. Weblogo of Sabc and CysA amino acid sequences. In the box were highlighted the conserved motifs common to ATP hydrolyzing subunits of ABC transporters: **Blue box**, motifs involved in binding and hydrolysis of ATP; **Orange box** motifs postulated to be involved in ATP hydrolysis and/or interaction with the membrane-spanning subunits.

```
SaSabc      mlpagraqalrssrdstacaakpgprs----vgsssrssfprrwvavqaldtsALEEQQQ    56
CrSabc      ma---sllaqttsrlgarpaaqagpvaqmapmasrvqpampsallpl haratttsv----    53
            *       *  :** .:  **: ** :    :.*  : ::*   : ::*  *::

SaSabc      QQQAAQLNGNGSSTAHQHRAAAAAGVSRPSGPTATLTDEDVNGMQLDGIQLEQQQQPTGL    116
CrSabc      acrAASIDKPVVYT---PRDSSQQSSNGAGEVSMSISSMDEVGPSYEGIITDAPTRPTGL    110
              :**.::     *    * ::  . .  .  : :::. *  * . :**  :   :****

SaSabc      YVRVRGMVKHFNTAKGVFKAVDGVDVDIPPSSICALLGPSGSGKTTLLRLVAGLELPTGG    176
CrSabc      YVRVRNMVKHFSTAKGLFRAVDGVDVDIEPSSIVALLGPSGSGKTTLLRLIAGLEQPTGG    170
            *****.*****.****:*:********* **** ****************:**** ****

SaSabc      KIYFDDLDATDLAVQDRQVGMVFQSYALFNHMTVSENIKFGLQVRKLPVDHDKRAADLLE    236
CrSabc      NIYFDDTDATNLSVQDRQIGFVFQSYALFNHKTVAENIKFGLEVRKLNIDHDKRVAELLA    230
            :***** ***:*:*****:*:********** **:*******:**** :*****.*:**

SaSabc      LVQLTGLGDRYPRQLSGGQRQRVALARALASNPRLLLLDEPFGALDAVVRKQLRAGLKEI    296
CrSabc      LVQLTGLGDRYPRQLSGGQRQRVALARALASNPRLLLLDEPFGALDAVVRKQLRTGLREI    290
            ******************************************************:**:**

SaSabc      VRSVGVTTIIVTHDQEEAFDLADQVVIFNRGLIEQSGSPNEIIKRPATPFVMGFVGDTNS    356
CrSabc      VRSVGVTTIIVTHDQEEAFDLADKVVVFNRGLVEQQGSPTEIIKRPRTPFIMKFVGETNV    350
            ***********************:**:*****:**.***.****** ***:* ***:**

SaSabc      VPAGCMLVRRSGFNPRLGKARVMFRPSDIRLSKEYVATL-DGQQVVTPATVNEAANMGWT    415
CrSabc      VPATSLLAKRMRFNT--SKTSVMFRPHDIKLFKTVPPESGEGALTTVGANVADKANLGWV    408
            *** .:*.:*  **   .*: ***** **:* *       :*  ... *.* : **:**.

SaSabc      MKYTLKFDDDVEVEFSVTRAQADKEFKLDVGQRIYVVVPPSAMMEFDETELGSAPII       472
CrSabc      VKYTLRFDDDVECELQLSRDQDEREYNLVVGSRVFVHVPHRTMMGFNASDVDSTPIV       465
            :****:****** *:.::* * ::*::* **.*::* **  :** *: :::.*:**:
```

Figure S5. Alignment between SaSabc and *C. reinhardtii* Sabc amino acid sequences: conserved motifs. Lowercase bold predicted transit peptide. **Bold fonts** conserved motifs common to ATP hydrolyzing subunits of ABC transporters: **Blue bold,** motifs involved in binding and hydrolysis of ATP; **Orange bold,** motifs postulated to be involved in ATP hydrolysis and/or interaction with the membrane-spanning subunits, according to Lindberg and Melis (2008) [25].

```
SaSBP     ------------------------------------------------------------   0
CrSBP     MSFLAPSLGVARGILEPASAARPPAHAAGHAPVLTSDRTGGPAANHDRPAGAPSPHAASL   60

SaSBP     ------------------------------------------------------------   0
CrSBP     TPSSSGQASQQGDPQRSQHQQAQRQDQQQSQSRSLQSHLITAATLLPALPPPPPGGNGDG   120

SaSBP     ----NPGPQPLALLAAANKEPLVLTVASFAVTKLAYVRLTKLFREKYLQEKGVDVRFRLT   56
CrSBP     DGGEAAGPQPLADVAA-QPPEVVLTLASFAVTKLAYVRVTRAFREWYERTKGVDVRFRLT   179
                ****** :** :   :***:************:*: *** * : **********

SaSBP     FAGSGVQARAVIDGLPADMVALALPLDVQKIADAGLLSANWQKGFPLGSVVCETTVALVV   116
CrSBP     FAASGVQARAVIDGLPADIVALALPLDLDKIVSAGLIRPDWRSAYPAASVVCETTVAFVV   239
          **.***************:********::**..***:  :*:..:* .*********:**

SaSBP     RPGNPKNIQSWEDLTQPGLQVIVANPKTAGVARWIFLALWGSKMKKGAAAAKEYITKVFD   176
CrSBP     RQGNPKNIRTWEDLTRAGVEVVLANPKTAGVARWIFLALWGAKMKKGNAAALAYVQRVFE   299
          * ******::*****: *::*::******************:***** ***  *: :**:

SaSBP     NVLVQPRDAREASDVFYRQRLGDVLLTYENEVVLTNQVYGPEKALPYVVPSPNVRIECPM   236
CrSBP     NVVVQPRDAREASDVFYKQKVGDVLLTYENEVILTNEVYGD-KALPYLVPSYNIRIECPL   358
          **:**************:*::***********:***:***  *****:*** *:*****:

SaSBP     ALVDKVLDARPAAAREAAHAFGKFCFTPEAQVEFGRVGFRTNRKLCKTPPAHLAGQPQIK   296
CrSBP     ALVDKVVDARGPEVREAASEFCRFLFTPAAQHEFARLGFRVNPRTCKEVAAQQTGLPPAN   418
          ******:***   .****  * :* *** ** **.*:***.* : **   *: :* *  :

SaSBP     MWTVDKELGGWGSAQRRFFDAGEILDQIQADVGARKAEARKAGKK---- 341
CrSBP     LWQVDKELGGWAAAQKKFFDAGAILDDIQSAVGKLRVEQRKAAQAAARR 467
          :* ********.:**::***** ***:**: **   :.* ***.:
```

Figure S6 Alignment between SaSBP and *C. reinhardtii* SBP amino acid sequences: conserved motifs. Lowercase bold predicted transit peptide. **Bold fonts** conserved region homologous to sulfate- and thiosulfate-binding proteins in cyanobacteria and other organisms according to Lindberg and Melis (2008) [25].

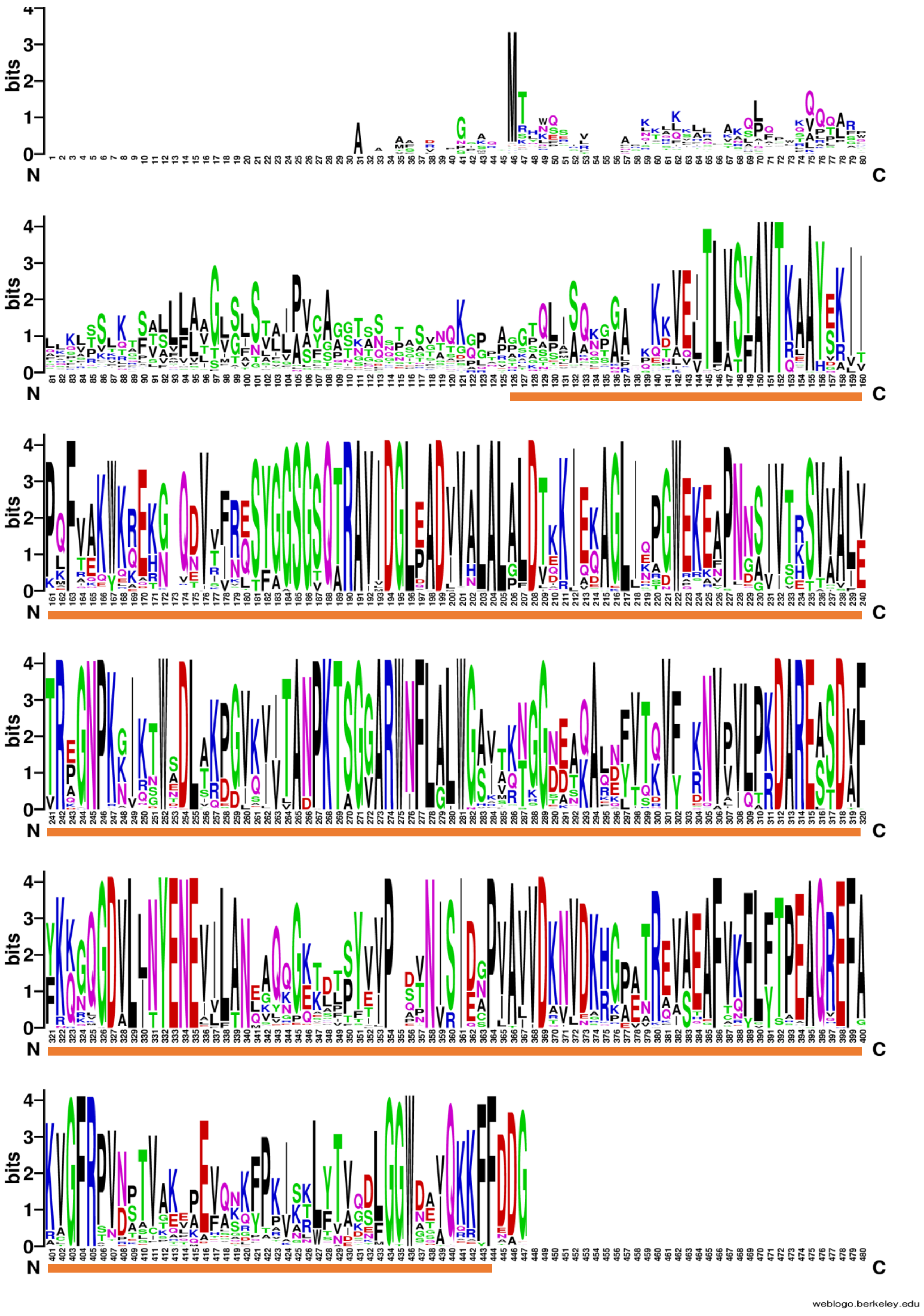

Figure S7. Weblogo of SBP and CysA amino acid sequences. Conserved region homologous to sulfate- and thiosulfate-binding (CysA) proteins and to SBP of eukaryotic organisms. **Orange line** denotes the position of SaSBP in the alignment.
